# Estimating bidirectional effects between social connectedness and mental health in adolescent students: Addressing biases due to endogeneity

**DOI:** 10.1371/journal.pone.0294591

**Published:** 2023-12-11

**Authors:** Chris Sakellariou

**Affiliations:** School of Social Sciences, Nanyang Technological University. Singapore, Singapore; University of Glasgow, UNITED KINGDOM

## Abstract

Research on the bidirectional relationship between social connectedness and health/mental health in adolescents is scarce, with most studies on adults. Some of the existing studies exploited the availability of longitudinal data to provide evidence of the *existence* of a causal relationship, either from social connectedness to health or establish a bidirectional relationship. There are at least two weaknesses associated with earlier research to assess the size of the effects. As acknowledged in the literature, one relates to attributing causality to empirical findings, due to well-known but inadequately addressed endogeneity biases. The other relates to failure to account for potentially important covariates, sometimes due to data limitations, or because such variables are not frequently used in sociological research. Existing research predominantly finds that the strongest path is from social connectedness to health/mental health, with effect estimates modest in size. I followed a quasi-experimental strategy by modelling adolescent students’ perceptions of social connectedness and mental health perceptions as potentially endogenous variables when estimating bidirectional effects. An instrumental variables (IV) modelling approach was followed, supplemented with a recently developed alternative approach to testing the exclusion restrictions of individual instruments. I exploited the rich information available in the PISA 2018 multi-country dataset, which allows for conditioning for a wide array of information on adolescent students’ personal circumstances, self-reported personality-related attributes, relationships with parents; and school characteristics. I found that (1) accounting for endogeneity biases is important; and (2) as opposed to findings reported in the literature, the dominant effect is from mental health perceptions to social connectedness for both male and female participants. The policy relevance of the findings is that adolescent mental health should be the primary focus of interventions, i.e., identifying and treating mental health symptoms as a primary intervention and as a precursor to improving the social connectedness of adolescents.

## 1. Introduction

The understanding that social relationships matter for mental health and health in general dates to the contributions of Émile Durkheim on socialisation in the late 19^th^ century [1]. Different studies have used different synonyms (often interchangeably) when referring to social connectedness, such as social networks, social integration, and social capital. Social connectedness (or social connection), which is subjective, is a broad concept which includes feelings or perceptions of belonging to a group of individuals, groups, or community [2]. On the other hand, a social network refers to relationships and interconnections in such relationships, hence is an objective reflection of one’s social environment. Social integration, a close synonym to social connectedness, includes sense of belonging and participation in social relationships [3]. Social capital is a term difficult to define precisely. Robison, Schmid, and Siles [4] proposed a definition which stresses sympathy, i.e., those who have sympathy for others provide social capital.

Interest in the relationship between various conceptualizations of social connectedness and health, including mental health, grew over the last few decades, especially around the turn of the millennium [5]. From the sociological and developmental psychology literature, social integration promotes mental health and health in general (such as mortality risk due to cardiovascular disease), and individuals with less social connections exhibit more psychological problems. Umberson and Montez [6] in their review of the ways social relationships influence health, cite behavioural, psychosocial, and physiological mechanisms. Social ties can influence health by influencing health habits such as exercise, diet, smoking, etc. Psychosocial explanations include mechanisms such as social support through the nurturing qualities of social relationships, which can positively affect mental health. Mental health, besides being a desirable outcome by itself, works in concert with other mechanisms influencing physical health [7]. Finally, physiological explanations refer to the potential of emotionally supportive social interactions to promote healthy development of immune, metabolic, and nervous systems, hence promoting long-term health [6].

### 1.1 Social connectedness in adolescent students

In adolescence, a critical period for hormonal changes potentially associated with symptoms of psychological distress, friendships develop involving emotional closeness which fulfils adolescents’ need for intimacy, a sense of belonging, and self-validation. It is the period when adolescents seek more independence from their families as they spend more time and develop intimate relationships with friends [8, 9]. Given that for adolescent students most of the friendships develop in school, feeling that one is well-integrated into friendship networks within the school environment and having a strong sense of belonging in school, is expected to have a beneficial effect on an adolescent student’s mental health.

It has been reported that students’ relationships with their parents, school peers, and teachers are linked to their psychological health and students with lower quantity and quality of peer relationships are at higher risk of developing psychological symptoms [10, 11]. Middle school students face significant challenges which can affect their mental health. With peer influence particularly pronounced during this period and the impact of family progressively fading, the school social environment plays an important role in regulating students’ mental health [12].

Several empirical studies have investigated the relationship between social connectedness and health in general, while empirical studies on the relationship between mental health and social connectedness in adolescent students, were designed to estimate effect sizes and were not narrowly focused with respect to the conceptualization of mental health, are few. For example, Diendorfer et al. [13] reviewed 33 studies, mostly in the US, aiming to investigate differences in social connectedness of children and early adolescents with mental disorders, versus those that develop neurotypically. About half of the studies were on autism spectrum disorder (ASD), several on attention deficit hyperactivity disorder (ADHD), and three on social anxiety disorder (SAD). Some other studies on adolescents focused on specific health outcomes in their association with social networks, such as alcohol consumption and suicide risk.

Studies with a broadly similar focus as this study but differing in methodological approach include Ueno [9], who used US National Longitudinal Study of Adolescent Health data and investigated whether those who are integrated into friendship networks have better mental health, as measured by the number of depressive symptoms. Findings from hierarchical linear models supported the general proposition that adolescents have fewer depressive symptoms when they are integrated into friendship networks at school, by finding that higher levels of integration were associated with fewer depressive symptoms. It was also found that adolescents with a strong sense of belonging in school had fewer depressive symptoms and that sense of belonging had a more direct effect on mental health symptoms than did the number of friends, supporting the argument that integration contributes to mental health by strengthening a sense of belonging. More recently, Santini et al. [14] used a large cross-sectional national sample of high school students in Denmark and multilevel regression analysis to assess associations between social disconnectedness in school and various mental health outcomes (including depression symptoms, anxiety symptoms, stress, and sleep problems). They found that social disconnectedness was positively associated with mental health problems and that this association was stronger with each addition in types of social disconnectedness.

### 1.2 Bidirectional causal pathways

While the public health literature has clearly established that social connectedness promotes mental and physical health, interest in a reverse causal relationship (deterioration in social connectedness because of mental health problems) is found mostly among clinicians. Studies intending to test bidirectional relationships and demonstrate the direction of causal effects between the two constructs are rare. While some studies on reciprocal relationships exist, evidence on adolescents is scarce.

Lester et al. [15] investigated the temporal association between feeling connected to school and mental health prior to and over the transition period from primary to secondary school. They used path analysis to model relationships between school connectedness, depression, and anxiety. Their findings suggest reciprocal relationships between connectedness and mental health, i.e., increased connectedness to school is associated with decreased depression and anxiety; conversely, increased depression and anxiety is associated with decreased connectedness to school. Ding et al. [16] investigated the relationship between community participation and mental health, using Australian nationally representative panel data. They intended to quantify the relationship between past mental health and future community participation, and vice versa. They found that better past well-being led to significant increases in informal social connectedness and civic engagement but decreases in political participation. Yu et al. [17] used data from the British Household Panel Survey and multilevel cross-lagged structural equation analysis to investigate the reciprocal relationship between social capital and health while controlling for demographic characteristics. Their findings suggest that social participation predicts subsequent change in perceived mental health. They also provided evidence of reverse effects, with both perceived mental and physical health appearing to be the dominant causal factor with respect to the prospective level of social networks. Saeri et al. [18] used a large longitudinal sample from the New Zealand Attitudes and Values survey and cross-lagged panel analysis to assess the bidirectional longitudinal relationship between social connectedness and mental health, controlling for baseline levels of both variables and demographics. Social connectedness was found to be a stronger and more consistent predictor of mental health than mental health was of social connectedness.

### 1.3 Objectives and contributions of the study

Despite the increased availability of longitudinal data that allow for tests of bidirectionality among constructs, there are at least two weaknesses associated with earlier research on the relationship between social connectedness on health, when the objective is deriving and comparing effect sizes. One relates to attributing causality to empirical findings, due to unobservables/omitted variables and associated biases. As assessed by Umberson and Montez [6]: [past research] *“…is vulnerable to misattributed causality…other factors not directly assessed may contribute to social ties and health behaviours in ways that produce observed links between the two*, *real or not…*” The endogeneity problem is well known, but inadequately addressed in most empirical studies. Although potential biases due to endogeneities are often pointed out as a limitation, researchers have little idea of the magnitude or even direction of the bias [19]. It requires using more specialised techniques to deal with complications due to unobservables. The other weakness generally encountered in empirical research on social connectedness and health, relates to failure to account for potentially important covariates, sometimes because they are not available in the dataset being used, or because such variables are not frequently used in sociological research. An example is psychological traits potentially associated with health behaviour as well as social connectedness. Similarly, when the social connectedness construct relates to adolescent students’ connections at school and outside the school, it would be useful to control for adolescents’ quality of connections with parents. Furthermore, when the research interest is in mental health, it is important to condition for overall health and related information. Finally, in research on adolescents, measures for adolescents’ various behaviours and habits such as the intensity of interest/use of information and communication technology (ICT), would be relevant as mediators of both psychological health and social relationships. For example, already lonely people may resort to using computers, which could result in fewer real-life contacts and relationships. Also, high-intensity computer use could have a negative impact on health, with symptoms such as headaches, body pains, sleep disorders, tiredness, etc., which in turn could trigger depressive feelings. However, computer use could also improve mental health through access to social support during times of depression [20].

Of the empirical approaches used which aim to uncover a causal relationship, especially while using observational data, some (e.g., multivariate regression, multilevel modelling, and propensity score matching), do not account for the possibility that one or more important confounders, observed or unobserved, could be another common cause of the outcome and the covariate of interest. There are, however, approaches which allow estimating a quasi-causal effect from data that arise from non-experimental research designs [21]. These include quasi-experimental strategies such as the potential outcomes/counterfactual theory of causality [22], the use of natural experiments, and instrumental variables (IV) approaches, which allow considerable control over omitted variables. A recent study [23] followed such an empirical strategy using cross-country European Social Survey data on adults and subjective general health as an outcome. They estimated an instrumental variable model and found that changes in social isolation influence subjective general health.

The specific objectives of this study are to model adolescent students’ perceptions of social connectedness and mental health as potentially endogenous covariates (by endogenous, I refer to a covariate appearing in a model which is correlated with unmeasured confounders which also affects the outcome), when estimating bidirectional effects, to account for potential biases due to endogeneity. For example, with respect to mental health, relevant unobserved confounders (the effects of person-to-person variation that have not been controlled for) include genetic and environmental factors. As reported in the relevant literature, the heritability estimates for anxiety and depression–among the most common emotional and behavioural problems in childhood and adolescence ‐ are 30–40%. Additional unobserved factors relate to individual-specific environmental influences, which sometimes start in the womb [24]. Similarly, with respect to social connectedness, unobservable selection factors may relate to the interaction between genetics (*genetic homophily*–the genotype of one individual student being predictive of the genotype of his/her friends) and the school environment. The school environment can influence the extent of friendship formations through the mechanism of homophilous relationships [25, 26]. Finally, an additional potential source of endogeneity is measurement/misreporting error associated with the covariate of interest. In the presence of classical (non-systematic) measurement error, effect estimates will generally be biased downwards, while with systematic measurement error, the direction of the bias cannot be easily predicted.

To address various possible potential sources of endogeneity of covariates of interest, an instrumental variables (IV) modelling approach was followed, supplemented with a recently developed alternative approach to deriving effect estimates in the presence of endogeneity and testing the exclusion restrictions of individual instruments. I also exploit the rich information available in the dataset, which allows for conditioning for a wide array of information on adolescent students’ personal circumstances, including self-assessed health, self-reported personality-related attributes, quality of relationships with parents; and school characteristics. Finally, models are estimated separately by gender, since the role of unobservables and associated biases may differ by gender, resulting in different effects by gender in any direction.

In this study, the social connectedness construct (defined in detail in section 2.2.1) encompasses adolescent students’ sense of belonging in school and satisfaction with their friendships in general. School connectedness (or sense of belonging in school) has been defined as ‘the extent to which students feel personally accepted, respected, included and supported by others in their environment’ [27].

The following research questions were investigated:

**RQ1**: Is perceived social connectedness (sense of belonging in school/satisfaction with friends) an endogenous covariate when estimating effects on mental health? Similarly, is perceived mental health an endogenous covariate when estimating effects in the opposite direction?

**RQ2**: Can reciprocal effects be established between social connectedness and mental health of adolescent students, and what is the direction of the dominant effect?

**RQ3**: Are there gender differences in findings associated with research questions 1 and 2?

## 2. Materials and methods

### 2.1 Data

I used the 2018 Program for International Student Assessment (PISA-2018) survey data on 15-year-old students. The 2018 version of PISA, besides the compulsory student and school questionnaires which contains information on students themselves and their family, included questionnaires on different aspects of students’ experiences in school, and students’ attitudes, dispositions, and beliefs. PISA 2018 also included an optional questionnaire on students’ well-being, with nine countries participating (Bulgaria, Georgia, Hong Kong, Ireland, Mexico, Panama, Serbia, Spain, and the United Arab Emirates). The well-being questionnaire focused on students’ perceptions of their physical and psychological health, subjective well-being indicators, social relationships, and activities outside of school. The well-being questionnaire assessed important factors for the well-being of adolescent students, both objective (such as health, living environments, and financial situation) and subjective (such as individual personality attributes). Among other well-being indicators of students as individuals (i.e., health, education, and psychological functioning), satisfaction with one’s body image is included in the well-being questionnaire. It also included an optional ICT familiarity questionnaire with questions on different aspects related to digital media and digital devices, internet access, and related questions.

#### 2.1.1 Measures

*Mental health*. Mental health disorders in adolescence are related to anxiety (such as irritability, tension, and difficulty sleeping), mood disorders and depression. Such disorders are not rare, for example in the US, one in 10 adolescents has a disorder that causes significant impairment, while one in five has a diagnosable mental health disorder that causes some degree of impairment [28]. Such disorders are frequently accompanied by physical aches and pains, such as stomach pains, or other somatic complaints. Children with chronic medical illnesses have increased rates of anxiety and depression compared to children without medical illnesses [29]. While it may be difficult to distinguish whether somatic complaints are a consequence of pre-existing mental disorders or vice versa, mental health problems should be addressed in children who have been treated for chronic somatic complaints.

In the Wellbeing questionnaire of PISA 2018, students were asked about the frequency of specific symptoms *(1 = Rarely or never; 2 = About every month; 3 = About every week; 4 = More than once a week; 5 = About every day)*. Symptoms relate to feeling depressed; feeling irritable or bad temper; feeling nervous; experiencing difficulties in getting to sleep; feeling dizzy; feeling anxious; experiencing headaches; and experiencing stomach aches. Based on responses, 16% of 15-year-old students reported that they felt depressed, 17% reported anxiety symptoms, and 21% had difficulties in getting to sleep, more than once a week.

Assessment of internal consistency of the responses to these items in the questionnaire by computing the Cronbach’s alpha statistic [30] using all questions about symptoms (as opposed to subsets of questions), indicated that the highest alpha value is derived from using all items (**α** = 0.88). I used principal component analysis to extract the information from the (reverse coded) item responses, to generate a continuous index taking negative and positive values, standardized at mean = 0 and SD = 1. More positive values indicate fewer mental health symptoms reported, i.e., better mental health.

*Social connectedness*: *Sense of belonging and satisfaction with friends*. In earlier research, various measures of social connectedness, i.e., the degree to which people are socially connected, have been used. Frequently used measures are *egocentric network* size and density, representing the number of others (*alters*) the focal person (*ego*) is connected to [9]. However, such measures only summarize direct social ties without accounting for adolescents’ perceptions of the quality of social connections. Given that most of the adolescent friendships develop in school, any measure of adolescent students’ social connectedness (i.e., sense of belonging to a group or community) should also capture their sense of belonging in school.

PISA-2018 contains the following items: (a) Satisfaction with friendship networks in general: *How satisfied are you with the friends you have*? (1 = Not at all satisfied; 2 = Not satisfied; 3 = Satisfied; 4 = Totally satisfied); (b) Six items related to a sense of connectedness/belonging in school: “*I make friends easily at school (reverse coded)*”; “*Other students seem to like me* (reverse coded)”; “*I feel like I belong at school* (reverse coded)”; “*I feel lonely at school*”; “*I feel awkward and out of place in my school*”; and “*I feel like an outsider (or left out of things) at school*” (1 = Strongly agree; 2 = Agree; 3 = Disagree; 4 = Strongly disagree); and (c) Number of friends (*At present*, *how many friends do you have*?), along with intensity of interaction with friends (“*How many days a week do you usually spend time with your friends right after school*?”; “*How often do you talk to your friends on the phone*, *send them text messages or have contact through social media*?”; and “*How easy is it for you to talk to your close friends about things that really bother you*?”).

The set of six items related to the sense of belonging in school exhibits high reliability for measuring the same construct (**α** = 0.806). This is also the case when these six items are combined with the item on satisfaction with friendship networks in general (**α** = 0.803). However, the items under (c) exhibit very poor internal consistency as a group (**α** = 0.107), suggesting that the number of friends and related items are standalone items. Furthermore, including these items in the same group of items on sense of belonging in school and satisfaction with friendship networks, reduces the internal consistency of the group considerably. For example, adding “number of friends” in the group containing items under (a) and (b), reduces Cronbach’s alpha statistic from 0.803 to 0.229.

Starting with the seven items under (a) and (b) (i.e., those measuring general satisfaction with friendship networks and sense of connectedness in school), I used principal component analysis to generate a continuous composite index taking negative and positive values, standardized at mean = 0 and S.D. = 1. More positive values indicate better social connectedness.

#### 2.1.2 Other covariates

In specifying the models, I investigated a wide variety of potential predictors of each outcome. When more than one related measure was available, I first investigated which one displays a closer association with the outcome variable. Covariates included in the model specification are:

Demographic and family characteristics (other than gender.

**Immigrant/minority status:** First or second-generation immigrant vs. native.

**Location**: Village, Town, City, or Big City.

**Overall health**: 15-year-olds assessed their health as “Excellent”, “Good”, “Fair”, or “Poor.

**Body Mass Index (BMI)**: Derived from questions on self-reported height in cm and weight in kgs. Using this index, I derived a categorical variable with BMI ranges for being underweight, of normal weight, overweight, and obese.

**Socioeconomic status** (standardised index). Derived from three variables related to parents’ highest level of education, parents’ highest occupational status, and home possessions.

Other student characteristics

**Interest in ICT** (standardised index): Derived from questions such as: “*It is very useful to have Social Networks on the Internet*”; “*I am really excited discovering new digital devices or applications*”; “*I really feel bad if no Internet connection is possible*”; and “*I like using digital devices*”.

**Hours of free time in a day:** Derived using information on school instruction time and student’s out-of-school study hours.

Psychological domain perceptions.

**Perceived body image** (standardized index): The body image continuous index contains negative and positive values and was derived in the survey from questions given on a four-point Linkert scale (from “Strongly disagree” to Strongly agree”): “*I like my look just the way it is*”; “*I consider myself to be attractive*”; “I *like my body*”; and “*I like the way my clothes fit me*”.

**Self-efficacy/Resilience** (standardised index): Derived in the survey from the following questions, “*I usually manage one way or the other*”, “*I feel proud that I have accomplished things*”, “*I feel that I can handle many things at a time*”, “*My belief in myself gets me through hard times*”, and “*When I am in a difficult situation*, *I can usually find my way out of it*.” Stronger agreement vs. disagreement to each question contributes to more positive vs. more negative values in the index.

**Fear of failure** (standardised index): Derived in the survey from answers to questions the following questions: “*When I am failing*, *I worry about what others think of me*”; “*When I am failing*, *I am afraid that I might not have enough talent*”; and “*When I am failing*, *this makes me doubt my plans for the future*”.

Perceptions of relationship with parents.

**Social connections with parents** (standardized index): Derived in the survey from questions capturing the student’s social connection to his or her parents, such as: “My parents help me as much as I need”, “My parents show that they care”; “*My parents try to understand my problems and worries*”, etc.

**Emotional support from parents** (standardised index): Derived in the survey from questions: “*My parents support my educational efforts and achievements*”; *“My parents support me when I face difficulties at school*”; and “*My parents encourage me to be confident*”.

School characteristics.

**School ownership:** Public, vs. Private (independent or government dependent) school.

**Single-sex vs. coeducational school:** Derived from variables on number of male and female students in school.

**School disciplinary climate** (standardised index): Disciplinary climate is measured by the extent to which students miss learning opportunities due to disruptive behaviour in the classroom, such as: “*Students don’t listen to what the teacher says*”; “*There is noise and disorder*”; “*The teacher has to wait a long time for students to quiet down*”; “*Students cannot work well*”; and “*Students don’t start working for a long time after the lesson begins*”.

### 2.2 Estimating causal bidirectional effects: Methodological approach

When using observational data, to meaningfully estimate models involving an outcome variable (y) and a vector of explanatory variables (x), assumes that: (a) vector x is uncorrelated with the error term in the equation to be estimated; (b) there is no simultaneity/reverse causation; and (c) variables in x are measured without error. When we expect violation of one or more of the above requirements ‐ in isolation or in combination with one or more of the covariates in x ‐ covariate endogeneity is a complication. For example, thinking in terms of the relationship between perceptions of social connectedness and mental health, the correlation between perceptions of social connectedness and the error term could arise because we have omitted one or more important covariates from our model which are correlated with perceptions of social connectedness; alternatively, it could be that perceptions of social connectedness and mental health symptoms are to some extent determined by the same unobserved factors. To address the objectives of the investigation, the methodological approach consists of three steps, as outlined below.

*Derive a plausible range of degree of endogeneity of the potentially endogenous covariate*: Using candidate instrument/s assumed to be relevant and valid (whose relevance and validity are later evaluated), I estimated Extended Regression Models (ERMs) with one covariate assumed to be endogenous. Such models, developed after Heckman’s [31] original work on causal inference models and related work [22], incorporate selection on both observables and unobservables.

The model to be estimated is as follows:

y=x1'β+w1β1+ε1 (1)

w1=x1'β2+x2γ1+ε2 (2)

The outcome in each direction of the relationship is determined by the potentially endogenous covariate w_1_, other covariates (vector x_1_), and error term *ε*_*1*_. Having identified candidates for instrument/s (x_2_), the potentially endogenous covariate, w_1_, is determined by a vector of variables that are correlated with w_1_, but not correlated with y, except through their effect on w_1_ (instrument vector x_2_), along with variables in vector x_1_, and error term *ε*_*2*_. Unobservables are represented by the error terms in the four equations.

The error covariance matrix is:

$$\Sigma \;=\;\left[\begin{array}{cc} \sigma^{2} & \rho_{12}\\ \rho_{12} & 1 \end{array}\right]$$

i.e., from this model, an estimate of the correlation in error terms (unobservables) between the two equations (ρ_12_) is derived, along with a confidence interval.

*Test exclusion restrictions at plausible degree of endogeneity*, *using kinky least-squares (KLS)*:

Excluded instruments need to be correlated with the endogenous regressor, but uncorrelated with the outcome. The exclusion restrictions of candidate instruments (individually, or in combination) can be tested using the recently developed *kinky least-squares* (KLS) approach [32, 33]. The main objective of kinky least-squares (KLS) is to provide an instrument-free alternative method of identifying coefficients in the presence of endogenous covariates. KLS yields: (a) statistical inference on the validity of exclusion restrictions regarding candidate external instruments, for a plausible range of endogeneity correlations. I used the point estimate and associated confidence interval of endogeneity correlations derived in step (1), and (b) instrument-free causal effect estimates for the range of values of plausible degree of endogeneity. These estimates come with substantially narrower confidence intervals compared to the IV estimates.

*Derive bidirectional effect estimates from IV estimation*: Using the proposed instruments, IV estimates of bidirectional effects were derived. When more than one instrument is available, in addition to the KLS-based exclusion restriction tests for individual instruments, conventional overidentification tests of instrument combination are reported (overidentification tests of all instruments).

### 2.3 Proposed instruments

In the direction from social connectedness to mental health (Model 1), the instrument used is the school-level average response (derived using the country-school identifier in the survey) to the question “*I feel like I belong in school*”, with responses ranging from strongly disagree to strongly agree. In the direction from mental health to social connectedness (Model 2), the main instrument set is based on responses to questions on how students felt when last attended math or language classes (*Bored; Challenged; Nervous/Tense; Motivated/Inspired*), with responses ranging from ‘Not at all, to Extremely). In the male regression, a combination of two instruments was used: feeling Nervous/Tense when attending a math class and feeling bored when attending a language class. In the female regression, feeling Nervous/Tense when attending a math class was used as a single instrument.

In model 2, an alternative ‐ school-level ‐ instrument was considered (see section 3.4). For males, the instrument is based on Yes/No responses at the school level to questions on how they felt the day before (i.e., “*accomplished something*”; “*were treated with respect*”; “*smiled or laughed*”; “*learn something interesting*”; “*had enough energy to get things done*”; “*overall*, *satisfied with their day*”). The instrument is the school average of: “*Yesterday*, *I had enough energy to get things done*”. For females, the instrument is based on responses about attitudes towards competition at school (“*I enjoy working in situations involving competition with others;* “*It is important for me to perform better than other people on a task*”; “*I try harder when I’m in competition with other people*”), with responses ranging from strongly disagree to strongly agree. The continuous ordered instrument used is the school mean of: “*It is important for me to perform better than other people on a task*”. Higher values indicate a more competitive school environment.

Given that the instruments used are ordered, there is a different local average treatment effect (LATE) parameter for every value of the instrument; hence, the linear IV estimator estimates a weighted average of local average treatment effects.

Instrument relevance and validity depend on the following main assumptions:

Relevance: The instrument and the endogenous covariate are sufficiently associated, either because of a causal association between the two or because the two share a common cause. In observational studies, it is assumed that there is an unmeasured causal instrument which is the common cause of the measured proxy instrument and the endogenous covariate [34]. In Model 1, the intuition is that the sense of belonging at the individual student level will be correlated to the sense of belonging at the group/school level. In Model 2, the more intense the feeling of nervousness/tension when attending a math class is expected to be negatively correlated with students’ mental health. With respect to the alternative instruments in Model 2, belonging to a group whose members are on average more satisfied with their daily lives at school, is expected to be positively correlated with the perceived mental health *of* an individual member. Finally, belonging in a more competitive school environment is expected to be negatively correlated with the perceived mental health of an individual student.

Independence: The instrument must be uncorrelated with the error term, i.e., the instrument does not share common causes with the outcome. Independence cannot be tested, since the error term is, by definition, unobservable.

*The exclusion restriction*: The instrument is independent of the outcome, after conditioning for additional covariates, i.e., the instrument affects the outcome only through its effect on the endogenous covariate.

An additional assumption needed to point-identify a causal effect is that of Monotonicity. This assumption is usually in the context of binary instruments [35, 36]. Deterministic monotonicity states that for each subject, the level of the treatment that a subject would take is a monotonic increasing function of the level of the instrument. This assumption is unlikely to hold in most cases. A weaker version of monotonicity, that of Stochastic Monotonicity, only requires a monotonic relationship between the IV and the probability of treatment, hence it is easier to satisfy. Small et al. [37], show that under stochastic monotonicity, the IV method identifies a weighted average of treatment effects.

Given the expectation that the instrument validity assumptions do not hold precisely, in section 3.4 (Further analysis), I implement the procedure suggested by Nevo and Rosen [38] which relaxes the key IV correlation assumption, by replacing the assumption of zero-correlation between the instrument and the unobserved error term with an assumption on the “sign” of the correlation, thus allowing for the construction of IV bounds under weaker-than-traditional assumptions.

### 2.4 Summary statistics

Table 1 contains the summary statistics of outcome variables and covariates by gender. Female participants reported significantly worse mental health complaints than male participants. From the related questionnaire items, the frequency of reported symptoms across all related questions for female participants is about 1.7 times higher than for male participants. On the other hand, perceptions of social connectedness do not differ significantly between male and female participants.

**Table 1:** Summary statistics by gender.

Characteristic	Male	Female	Male-Female Diff.
Mental health index (mean)	0.285	-0.159	*
Social connections index (mean)	-0.044	-0.076	-
Village/small town (%)	28.66	27.17	-
Town (%)	24.40	21.68	*
City (%)	26.61	28.35	*
Large city (%)	20.32	22.80	*
Immigrant (%)	12.95	13.24	-
SES (index mean)	-0.805	-0.858	-
Health: Excellent (%)	47.81	33.37	*
Health: Good (%)	42.00	49.44	*
Health: Fair (%)	8.96	16.05	*
Health: Poor (%)	1.22	1.44	-
BMI range: Normal weight (%)	78.26	77.83	-
BMI range: Underweight (%)	4.69	3.57	*
BMI range: Overweight (%)	11.75	13.94	*
BMI range: Obese (%)	5.30	4.68	*
Body image satisfaction (index mean)	0.199	0.030	*
Interest in ICT (index mean)	-0.043	-0.012	-
Hours of free time per day (mean)	7.04	6.73	*
Resilience/self-efficacy (index mean)	0.254	0.254	-
Fear of failure (index mean)	-0.054	0.057	*
Parents’ emotional support (index mean)	-0.085	0.128	*
Social connections with parents (index mean)	-0.205	-0.112	*
Public school (%)	83.34	81.51	*
Single sex school (%)	1.52	2.66	*
School disciplinary climate (index mean)	-0.112	0.014	*

Note: * indicates male-female difference is significant at the 5% level or lower.

Males assess their health as “excellent” at substantially higher rates than females (48% vs. 33%), while a higher proportion of females assess their health as “fair” (16% vs. 9%). Females reported significantly lower body image satisfaction than males. Females reported a higher fear of failure, while males reported substantially less emotional support from, and worse social connections with parents, compared to females. Finally, based on their experiences in school, males reported a worse disciplinary climate in school (such as noise and disorder in the classroom).

### 2.5 Instrument testing

When more than one instrument was available, instrument relevance and exclusion restrictions were assessed using conventional tests from IV estimation. In addition, I used the *kinky least-squares* (KLS) approach, which yields statistical inference on the validity of exclusion restrictions regarding candidate external instrument/s (single, or as a set) when a plausible range of endogeneity correlations is available, whereas these restrictions were always considered non-testable. In the IV framework, if the model is ‘just identified’ (i.e., one instrument for one endogenous regressor) the exclusion restriction cannot be tested; IV estimators assume that the direct effect of the instrument on the outcome is 0. Even in ‘overidentified’ models (i.e., at least two instruments for one endogenous regressor), tests of overidentifying restrictions rely on the (untested) assumption that at least as many instruments are validly excluded from the model as endogenous regressors [39]. The KLS approach (implemented using the *kinkyreg* module in Stata) supplements standard IV inference by allowing assessment of the validity of previously untestable “just-identifying” exclusion restrictions [33]. In other words, if instruments are available, this approach allows for sensitivity checks for IV-based estimation. In this sense, it supplements IV estimation.

Instrument testing using KLS requires prior knowledge of a plausible range of degree of endogeneity (endogeneity correlations) of the endogenous covariate; to derive estimates of plausible endogeneity correlations, I estimated Extended Regression Models (ERMs) with one endogenous covariate (using the *eregress* module in Stata), and the same set of instruments as in the IV regressions. Given a set of instruments, ERMs produce coefficient estimates which closely mimic those from the IV estimation; in addition, they produce estimates and confidence intervals of endogeneity correlations (correlations of error terms between the outcome equation and the equation determining the potentially endogenous regressor), which served the purpose of a plausible range of endogeneity correlations when testing exclusion restrictions using KLS.

## 3. Results

Multiple (OLS) regression estimates (column 1) and IV regression estimates (column 2) use the survey weights, along with 80 BRR replication weights for correctly calculating standard errors. Given that the nine countries represented in the data are heterogeneous with respect to geography, culture, etc., to account for such cross-country differences, all regressions control for country fixed-effects. KLS estimates of the effect of social connectedness on mental health (column 3) are unweighted.

### 3.1 Model 1: From social connectedness to mental health

Table 2 contains the OLS and IV results by gender, with the social connectedness composite as a potentially endogenous covariate. The top panel contains the coefficient estimates, while the middle panel contains the first-stage results (coefficient estimates of the model determining social connectedness). The bottom panel gives the F-statistic for excluded instruments and test of endogeneity of social connectedness. With a single instrument, the exclusion restrictions were tested within the KLS framework, for a plausible range of degree of endogeneity of the social connectedness composite derived from estimating ERMs.

**Table 2:** From social connectedness to mental health: Effect estimates by gender.

Outcome: Mental Health (stand.)	MALES			FEMALES		
	OLS	IV	KLS	OLS	IV	KLS
**Social Connectedness (stand. index)**	**0.131**	0.195	**0.207**	**0.096**	**0.284**	**0.285**
	(0.016)	(0.119)	(0.007)	(0.017)	(0.112)	(0.008)
Town (vs. Village)	**-0.082**	**-0.091**		**-0.105**	**-0.101**	
	(0.037)	(0.040)		(0.042)	(0.044)	
City (vs. Village)	-0.076	**-0.078**		**-0.087**	-0.053	
	(0.040)	(0.040)		(0.040)	(0.046)	
Large city (vs. Village)	-0.070	-0.075		**-0.155**	**-0.133**	
	(0.041)	(0.042)		(0.044)	(0.047)	
Immigrant	-0.000	0.012		0.063	**0.100**	
	(0.027)	(0.034)		(0.038)	(0.043)	
SES (stand. index)	**-0.052**	**-0.051**		**-0.075**	**-0.080**	
	(0.012)	(0.012)		(0.013)	(0.014)	
Health: Good (vs. Excellent)	**-0.240**	**-0.235**		**-0.278**	**-0.257**	
	(0.027)	(0.032)		(0.031)	(0.035)	
Health: Fair (vs. Excellent)	**-0.579**	**-0.563**		**-0.858**	**-0.818**	
	(0.062)	(0.073)		(0.051)	(0.058)	
Health: Poor (vs. Excellent)	**-1.40**	**-1.37**		**-1.25**	**-1.23**	
	(0.114)	(0.132)		(0.163)	(0.173)	
Underweight (vs. Normal weight)	-0.038	-0.032		**-0.229**	**-0.248**	
	(0.070)	(0.073)		(0.074)	(0.083)	
Overweight (vs. Normal weight)	0.028	0.021		-0.061	-0.025	
	(0.041)	(0.041)		(0.047)	(0.052)	
Obese (vs. Normal weight)	0.081	0.074		0.046	0.071	
	(0.061)	(0.063)		(0.073)	(0.078)	
Body image satisfaction (stand. index)	0.023	0.011		**0.073**	**0.048**	
	(0.015)	(0.025)		(0.016)	(0.022)	
Interest in ICT (stand. index)	**-0.104**	**-0.106**		**-0.160**	**-0.175**	
	(0.014)	(0.016)		(0.017)	(0.021)	
Hours of free time per day	-0.007	-0.011		**0.016**	0.013	
	(0.008)	(0.010)		(0.008)	(0.008)	
Resilience/self-efficacy (stand. index)	-0.015	-0.027		0.020	-0.019	
	(0.014)	(0.029)		(0.016)	(0.030)	
Fear of failure (stand. index)	**-0.111**	**-0.104**		**-0.163**	**-0.152**	
	(0.015)	(0.018)		(0.016)	(0.017)	
Emotional support from parents (stand. index)	0.007	0.002		-0.008	-0.024	
	(0.016)	(0.019)		(0.019)	(0.022)	
Social connections with parents (stand. index)	**0.087**	**0.077**		**0.137**	**0.117**	
	(0.015)	(0.019)		(0.018)	(0.022)	
Public school	-0.011	-0.001		**-0.126**	**-0.107**	
	(0.030)	(0.032)		(0.034)	(0.036)	
Single sex school	**-0.223**	**-0.197**		-0.073	-0.055	
	(0.035)	(0.056)		(0.072)	(0.074)	
School disciplinary climate (stand. index)	**0.089**	**0.083**		**0.096**	**0.073**	
	(0.014)	(0.016)		(0.015)	(0.019)	
Constant	**0.594**	**0.648**		**0.304**	**0.380**	
	(0.080)	(0.135)		(0.087)	(0.109)	
R^2^	0.195	0.261		0.266	0.240	
F-statistic		38.6			63.3	
[p-value]		[0.000]			[0.000]	
**First stage IV: Social Connectedness**						
**School mean: “I feel like I belong at school”**	-	**-0.332**			**-0.321**	
		(0.044)			(0.036)	
Town (vs. Village)		**0.127**			-0.010	
		(0.040)			(0.037)	
City (vs. Village)		0.036			**-0.162**	
		(0.042)			(0.038)	
Large city (vs. Village)		0.044			**-0.087**	
		(0.046)			(0.041)	
Immigrant		**-0.149**			**-0.183**	
		(0.034)			(0.035)	
SES (stand. index)		0.019			**0.029**	
		(0.013)			(0.012)	
Health: Fair (vs. Poor)		**-0.165**			**-0.140**	
		(0.030)			(0.030)	
Health: Good (vs. Poor)		**-0.303**			**-0.245**	
		(0.060)			(0.045)	
Health: Excellent (vs. Poor)		**-0.495**			-0.164	
		(0.202)			(0.105)	
Underweight (vs. Normal weight)		-0.057			0.125	
		(0.077)			(0.075)	
Overweight (vs. Normal weight)		0.018			**-0.188**	
		(0.042)			(0.042)	
Obese (vs. Normal weight)		0.116			-0.078	
		(0.065)			(0.057)	
Body image satisfaction (stand. index)		**0.159**			**0.125**	
		(0.017)			(0.016)	
Interest in ICT (stand. index)		**0.056**			**0.099**	
		(0.017)			(0.017)	
Hours of free time per day		**0.053**			**0.015**	
		(0.008)			(0.007)	
Resilience/self-efficacy (stand. index)		**0.203**			**0.207**	
		(0.017)			(0.015)	
Fear of failure (stand. index)		**-0.087**			**-0.060**	
		(0.017)			(0.015)	
Emotional support from parents (stand. index)		**0.091**			**0.078**	
		(0.018)			(0.017)	
Social connections with parents (stand. index)		**0.098**			**0.101**	
		(0.016)			(0.016)	
Public school		**-0.076**			**-0.078**	
		(0.036)			(0.032)	
Single sex school		**-0.313**			-0.100	
		(0.035)			(0.060)	
School disciplinary climate (stand. index)		**0.056**			**0.110**	
		(0.015)			(0.014)	
Constant		-0.135			**0.227**	
		(0.136)			(0.015)	
Relevance of excluded instrument						
F-statistic for excluded instruments		57.2			81.4	
Test of endogeneity of social connectedness						
Chi-sq. statistic		0.308			2.89	
[p-value]		[0.579]			[0.089]	
N	23,458	23,458	23,458	25,706	25,706	25,706

OLS and IV estimates from weighted regressions with robust standard errors. Bold indicates statistical significance at the 5% level.

KLS (unweighted) estimates at the point estimate of degree of endogeneity of social connections.

The estimates of the effect of better social connections on mental health from multiple linear (OLS) regressions are small; one SD increase in the social connectedness composite is associated with about 0.1 SD increase in the mental health composite. The small difference in estimates by gender is not statistically significant. These effect estimates are of similar magnitude to those from other studies which rely on longitudinal data but do not consider endogeneity biases [18].

Columns 2 and 3 in Table 2 report estimates adjusted for endogeneity of social connectedness perceptions from two approaches, one instrument based (IV) and the other instrument-free (KLS). Given that KLS estimates are unweighted, they come with much smaller standard errors compared to the IV and the OLS estimates, but since they are unweighted, they are inconsistent. The point estimates and 95% confidence intervals of endogeneity correlations are -0.067 [95% CI: -0.13, 0.01] for males and -0.131 [95% CI: -0.20, -0.04] for females.

**Fig 1:**
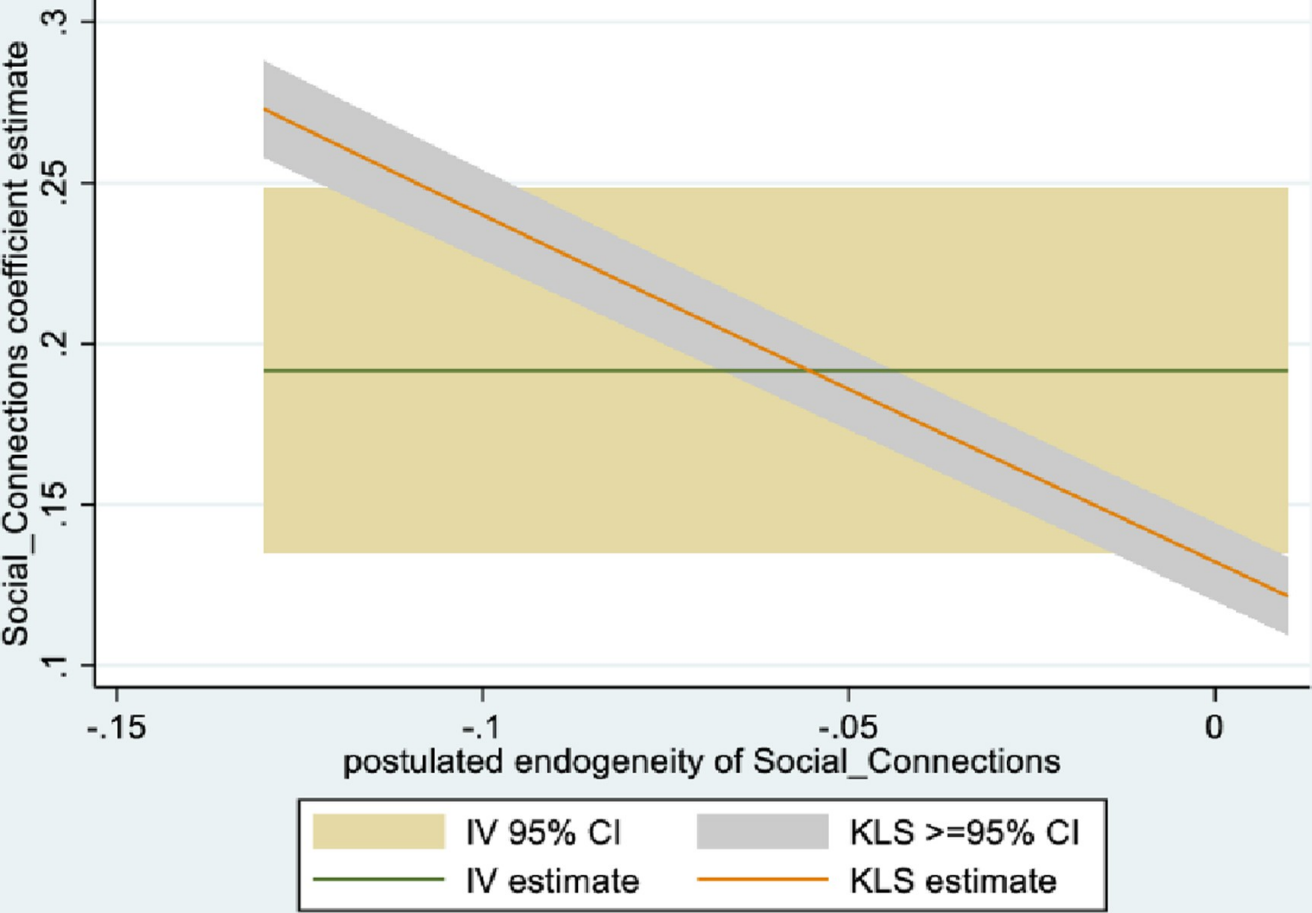
Model 1 KLS estimates: Males.

**Fig 2:**
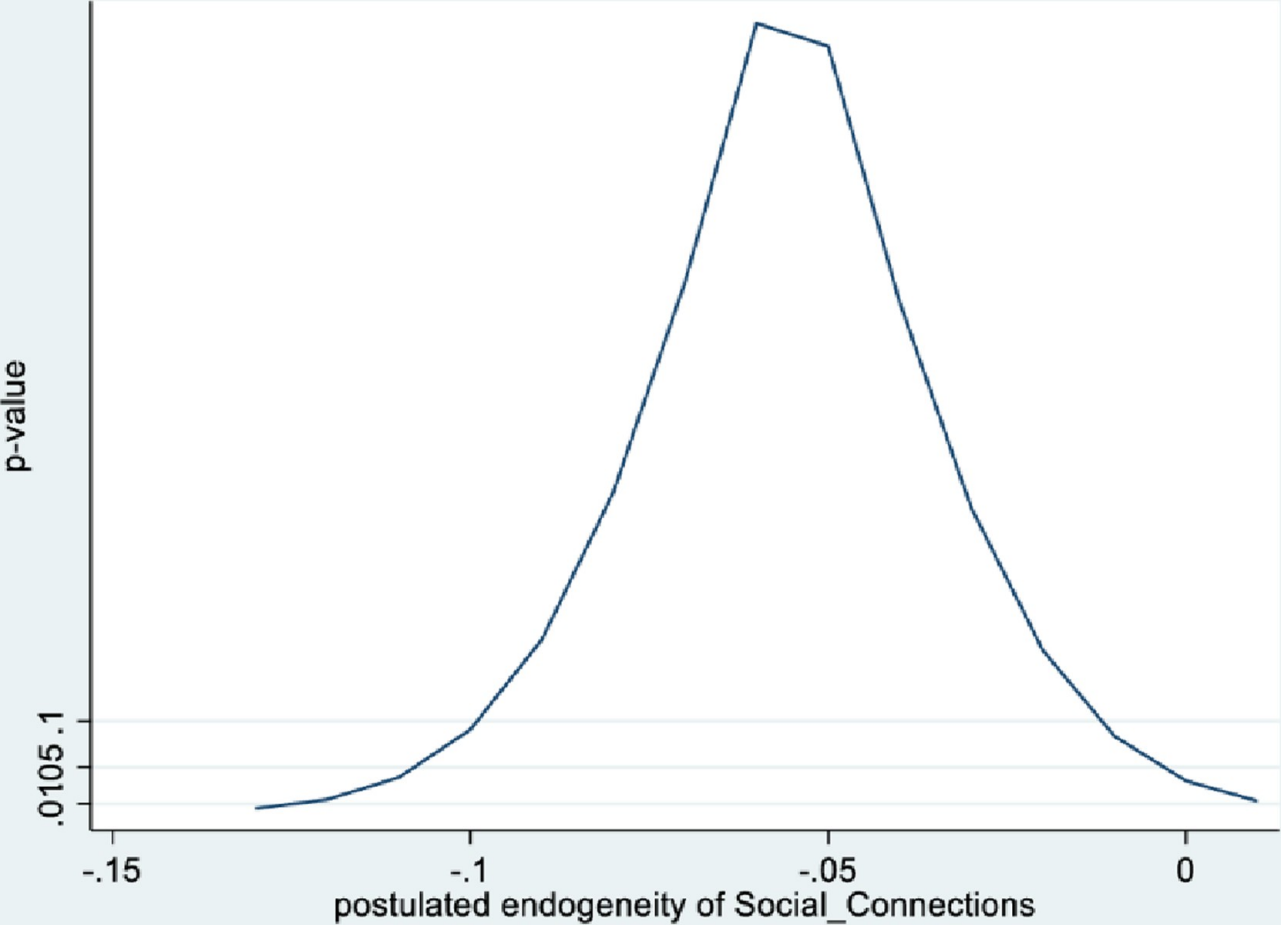
Model 1 exclusion restriction test: Males Endogeneity correlation point estimate: -0.066.

**Fig 3:**
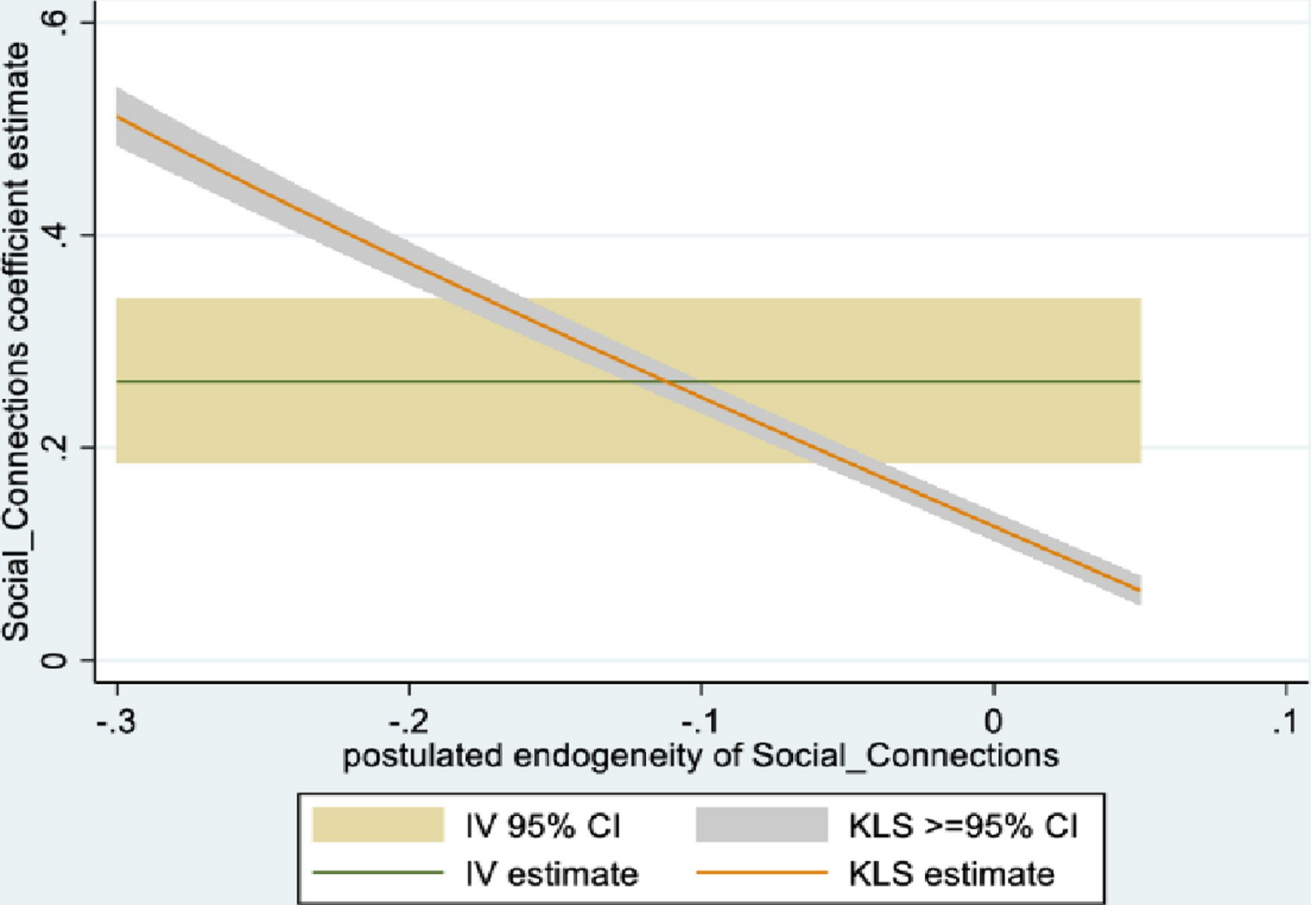
Model 1 KLS estimates: Females.

**Fig 4:**
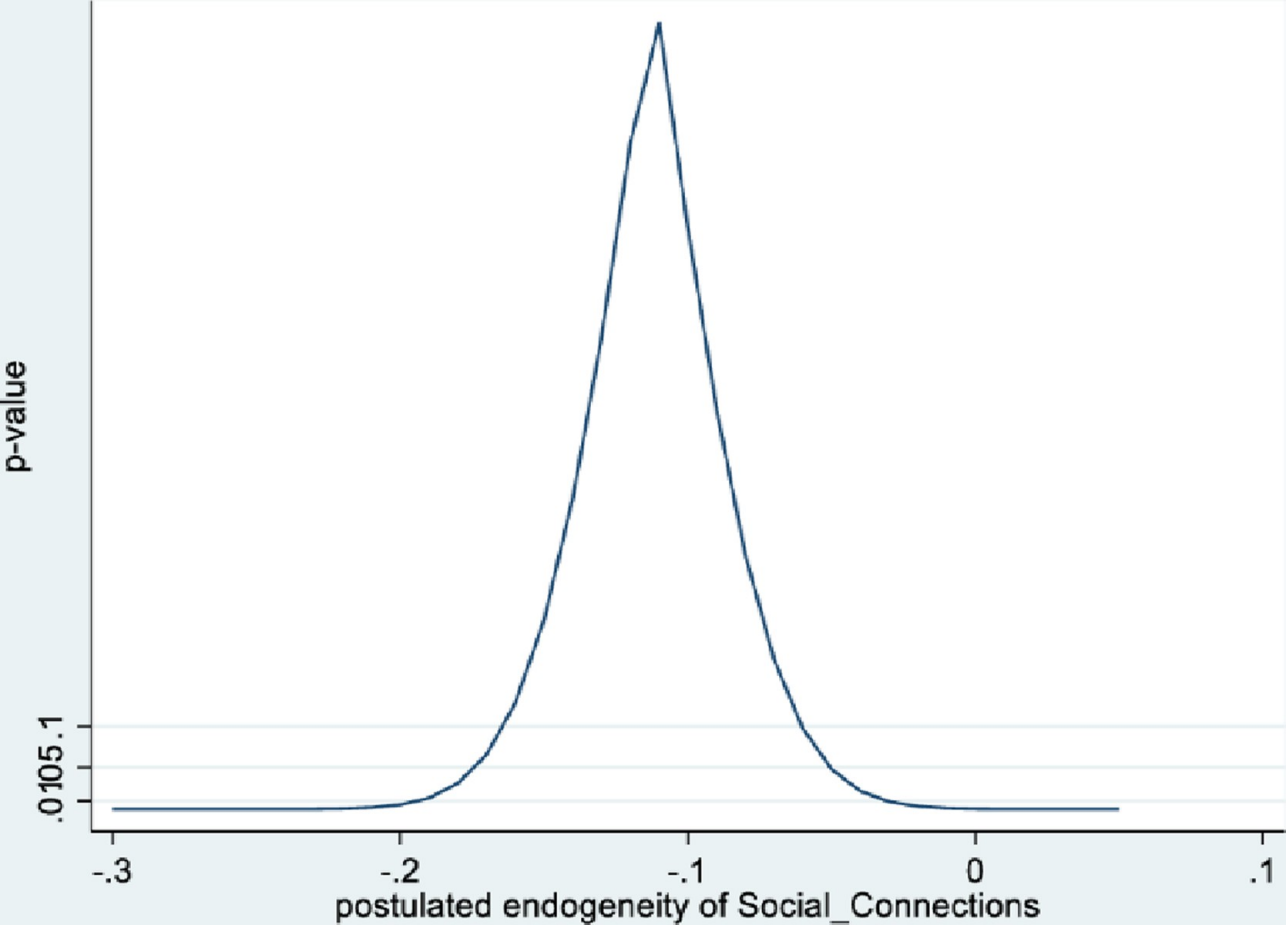
Model 1 exclusion restriction test: Females Endogeneity correlation point estimate: -0.13.

Figs 1 and 2 depict the KLS estimates and exclusion restriction tests for males, while Figs 3 and 4 are the corresponding figures for females. Figs 2 and 4 depict the p-values for Wald tests at various values of the postulated endogeneity correlations. For males, the hypothesis that the instrument is validly excluded from the model is accepted for the [-0.11, 0] range of endogeneity correlations, while for females the corresponding range of acceptance range is [-0.176, -0.05]. At the point estimates of -0.067 and -0.131 for males and females respectively, the hypothesis is accepted at high p-values.

The IV (2SLS) estimates of the effect of social connectedness on mental health are somewhat larger than the OLS estimates, but as is usually the case, less precisely estimated (β = 0.20; 95% CI = [-0.038, 0.43] for males and β = 0.28; 85% CI = [0.066, 0.50] for females). These estimates are essentially identical to the KLS estimates (0.21 for males and 0.28 for females). While the IV estimates are somewhat larger for females compared to males, the difference is not statistically significant.

Endogeneity tests accept the null hypothesis of exogeneity of the social connectedness composite in the male sample. In the female sample, there is evidence of endogeneity, with an associated p-value of 0.09. Finally, tests of instrument relevance indicate that the instrument is not weak. Using the rule of thumb, the first stage F-statistics, at 57 and 81 respectively for males and females, far exceeds 10.

### 3.2 Model 2: From mental health to social connectedness

Table 3 reports results in the direction from mental health to social connectedness. IV estimates are based on instruments related to how students felt when they last attended a math/language class. Results from using alternative instruments are discussed in section 3.4 (Further analysis). Multiple linear regression estimates (column 1) are small and similar to those in the opposite direction, with effect sizes ranging from 0.09 to 0.16 for female and male participants respectively. The gender difference in associations is statistically significant at the 5% level.

**Table 3:** From mental health to social connectedness: Effect estimates by gender.

Outcome: Social Connectedness (stand. index)	MALES			FEMALES		
	OLS	IV	KLS	OLS	IV	KLS
**Mental Health (stand. index)**	**0.156**	**0.583**	**0.641**	**0.085**	**0.428**	**0.494**
	-0.024	-0.151	(0.022)	(0.018)	(0.119)	(0.013)
Town (vs. Village)	**0.123**	**0.161**		-0.002	-0.011	
	(0.056)	(0.053)		(0.060)	(0.062)	
City (vs. Village)	0.039	0.081		**-0.162**	**-0.138**	
	(0.056)	(0.056)		(0.067)	(0.065)	
Large city (vs. Village)	0.038	0.086		-0.094	-0.053	
	(0.061)	(0.061)		(0.064)	(0.054)	
Immigrant	**-0.168**	**-0.180**		**-0.213**	**-0.239**	
	-0.04	-0.041		-0.04	-0.041	
SES (stand. index)	0.028	**0.047**		0.035	**0.051**	
	(0.019)	(0.019)		(0.023)	(0.023)	
Health: Good (vs. Excellent)	**-0.117**	-0.035		**-0.109**	-0.002	
	(0.043)	(0.047)		(0.041)	(0.052)	
Health: Fair (vs. Excellent)	**-0.207**	0.051		**-0.157**	0.103	
	(0.075)	(0.107)		(0.061)	(0.120)	
Health: Poor (vs. Excellent)	-0.277	0.532		-0.056	0.304	
	(0.280)	(0.318)		(0.123)	(0.237)	
Underweight (vs. Normal weight)	-0.046	-0.036		0.111	0.117	
	(0.075)	(0.101)		(0.179)	(0.240)	
Overweight (vs. Normal weight)	0.021	0.029		**-0.177**	**-0.065**	
	(0.049)	(0.058)		(0.087)	(0.071)	
Obese (vs. Normal weight)	0.096	0.006		-0.113	-0.178	
	(0.070)	(0.105)		(0.094)	(0.097)	
Body image satisfaction (stand. index)	**0.152**	**0.135**		**0.119**	**0.099**	
	(0.022)	(0.030)		(0.024)	(0.030)	
Interest in ICT (stand. index)	**0.070**	**0.113**		**0.106**	**0.186**	
	(0.020)	-0.025		-0.026	-0.036	
Hours of free time per day	**0.055**	**0.059**		0.012	0.001	
	(0.012)	(0.013)		(0.011)	(0.015)	
Resilience/self-efficacy (stand. index)	**0.205**	**0.189**		**0.210**	**0.166**	
	(0.018)	(0.019)		(0.025)	(0.026)	
Fear of failure (stand. index)	**-0.067**	-0.025		-0.040	-0.003	
	(0.019)	(0.030)		(0.021)	(0.029)	
Emotional support from parents (stand. index)	**0.090**	**0.084**		**0.082**	**0.120**	
	(0.020	(0.022)		(0.028)	(0.028)	
Social connections with parents (stand. index)	**0.085**	0.020		**0.089**	0.013	
	(0.023)	(0.031)		(0.023)	(0.029)	
Public school	**-0.081**	-0.060		-0.088	-0.035	
	(0.041)	(0.049)		(0.048)	(0.050)	
Single sex school	**-0.329**	**-0.204**		-0.084	-0.050	
	(0.075)	(0.091)		(0.060)	(0.073)	
School disciplinary climate (stand. index)	**0.041**	-0.0000		**0.103**	**0.056**	
	(0.020)	(0.023)		(0.028)	(0.029)	
Constant	**-0.953**	**-1.18**		**-0.453**	**-0.450**	
	(0.107)	(0.147)		(0.119)	(0.142)	
R^2^	0.267	0.163		0.282	0.195	
F-statistic		60			40.9	
[p-value]		[0.000]			[0.000]	
**First stage IV: Mental Health**						
**Attending a math class, you felt: Nervous/Tense**		**-0.158**			**-0.168**	
		(0.020)			(0.021)	
**Attending a test language class, you felt: Bored**		**-0.064**			**-**	
		(0.024)				
Town (vs. Village)		-0.066			-0.084	
		(0.060)			(0.070)	
City (vs. Village)		-0.057			-0.105	
		(0.070)			(0.068)	
Large city (vs. Village)		-0.061			**-0.163**	
		(0.069)			(0.059)	
Immigrant (vs. Village)		-0.015			0.064	
		(0.043)			(0.043)	
SES (stand. index)		**-0.042**			**-0.066**	
		(0.013)			(0.020)	
Health: Good (vs. Excellent)		**-0.269**			**-0.276**	
		(0.033)			(0.046)	
Health: Fair (vs. Excellent)		**-0.55**			**-0.783**	
		(0.085)			(0.058)	
Health: Poor (vs. Excellent)		**-0.1.45**			**-1.25**	
		(0.183)			(0.142)	
Underweight (vs. Normal weight)		0.003			-0.217	
		(0.088)			(0.172)	
Overweight (vs. Normal weight)		0.021			-0.072	
		(0.069)			(0.074)	
Obese (vs. Normal weight)		0.11			0.027	
		(0.080)			(0.117)	
Body image satisfaction (stand. index)		0.045			**0.085**	
		(0.024)			(0.019)	
Interest in ICT (stand. index)		-0.075			**-0.154**	
		(0.019)			(0.025)	
Hours of free time per day		-0.005			0.017	
		(0.014)			(0.013)	
Resilience/self-efficacy (stand. index)		-0.013			**0.046**	
		(0.017)			(0.019)	
Fear of failure (stand. index)		**-0.109**			**-0.142**	
		(0.024)			(0.021)	
Emotional support from parents (stand. index)		0.022			-0.014	
		(0.021)			(0.027)	
Social connections with parents (stand. index)		**0.087**			**0.141**	
		(0.021)			(0.020)	
Public school		-0.042			**-0.128**	
		(0.046)			(0.041)	
Single sex school		**-0.211**			-0.091	
		(0.060)			(0.148)	
School disciplinary climate (stand. index)		**0.062**			**0.104**	
		(0.021)			(0.020)	
Constant		0.897			**0.502**	
		(0.104)			(0.144)	
Relevance of excluded instrument						
F-statistic for excluded instruments		46.5			89.5	
Overidentification test of all instruments						
Hansen’s J statistic		0.250			-	
[p-value]		[0.610]				
Test of endogeneity of mental health						
Chi-sq. statistic		16.2			11.52	
[p-value]		[0.000]			[0.001]	
N	23,513	20,610	20,610	25,761	23,522	23,522

OLS and IV estimates from survey regressions with 80 BRR replications. Bold indicates statistical significance at the 5% level.

KLS (unweighted) estimates at the point estimate of degree of endogeneity of social connections.

However, IV estimates (column 2) are about 5 times larger than the OLS estimates (β = 0.58; 95% CI = [0.28, 0.88] for males β = 0.43; 95% CI = [0.19, 0.66] for females) and the gender difference in effect size is not statistically significant. Effect estimates from KLS present a similar picture (0.64 for males and 0.49 for females). These large effect sizes reflect the strong evidence of endogeneity of mental health perceptions. Point estimates and confidence intervals of error term correlations between the outcome and the equation modelling mental health perceptions are strongly negative (-0.37 [95% CI: -0.55, -0.15] for males and -0.32 [95% CI: -0.39, -0.25] for females). The negative error term correlations indicate that unobservables which tend to improve mental health perceptions occur with unobservables which tend to worsen social connectedness perceptions.

**Fig 5:**
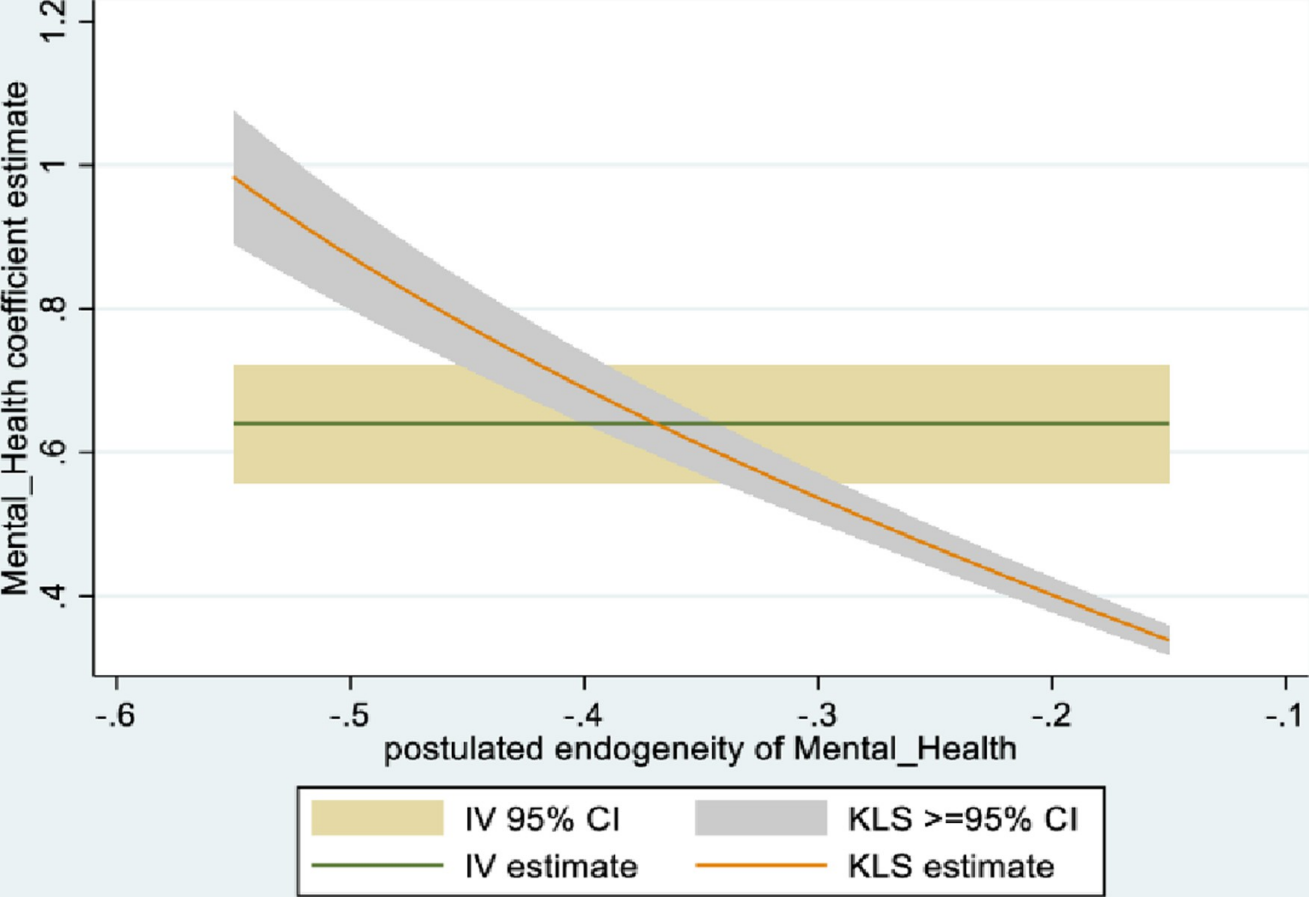
Model 2 KLS estimates: Males.

**Fig 6:**
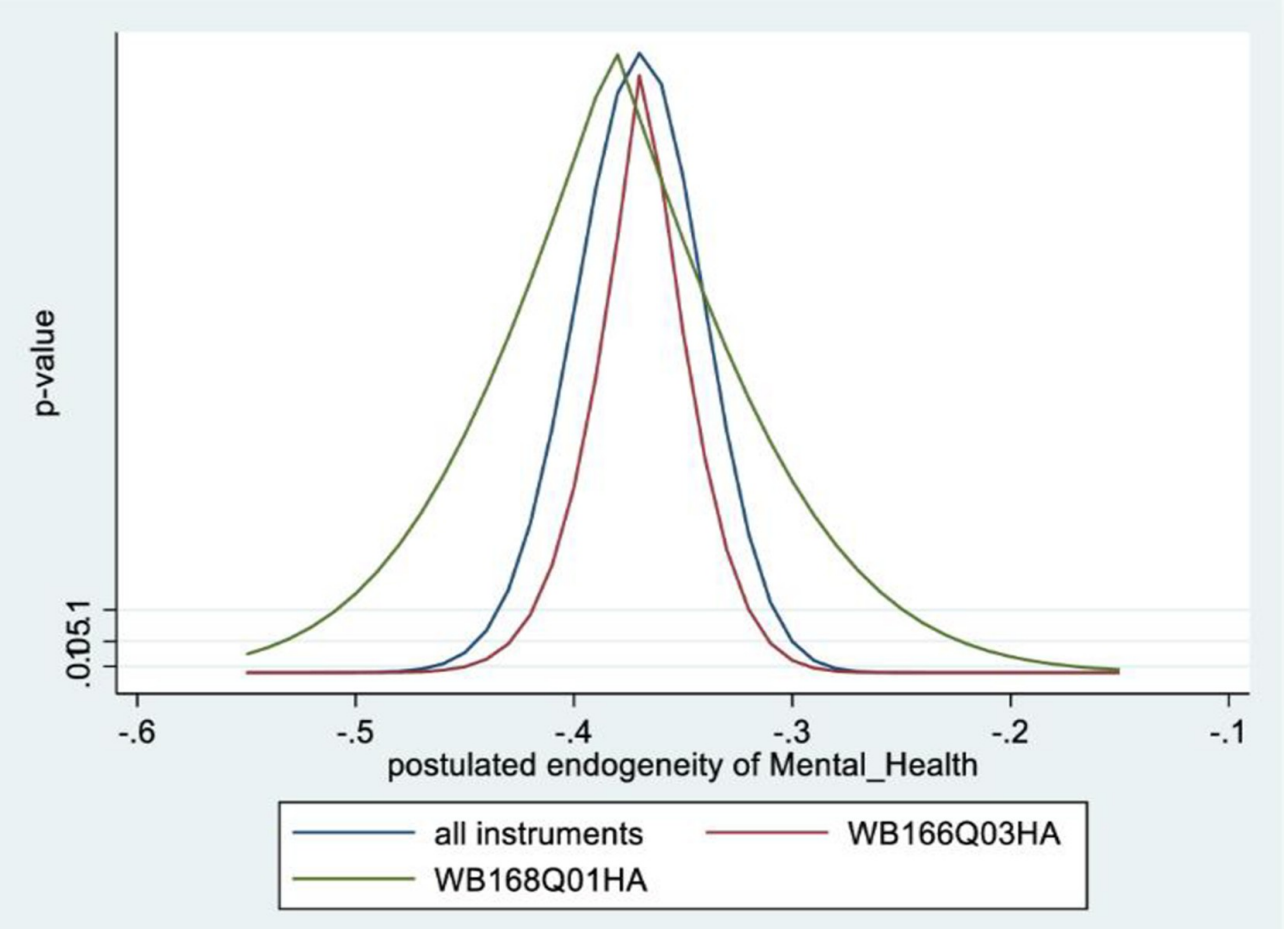
Model 2 exclusion restriction test: Males Endogeneity correlation point estimate: -0.37.

**Fig 7:**
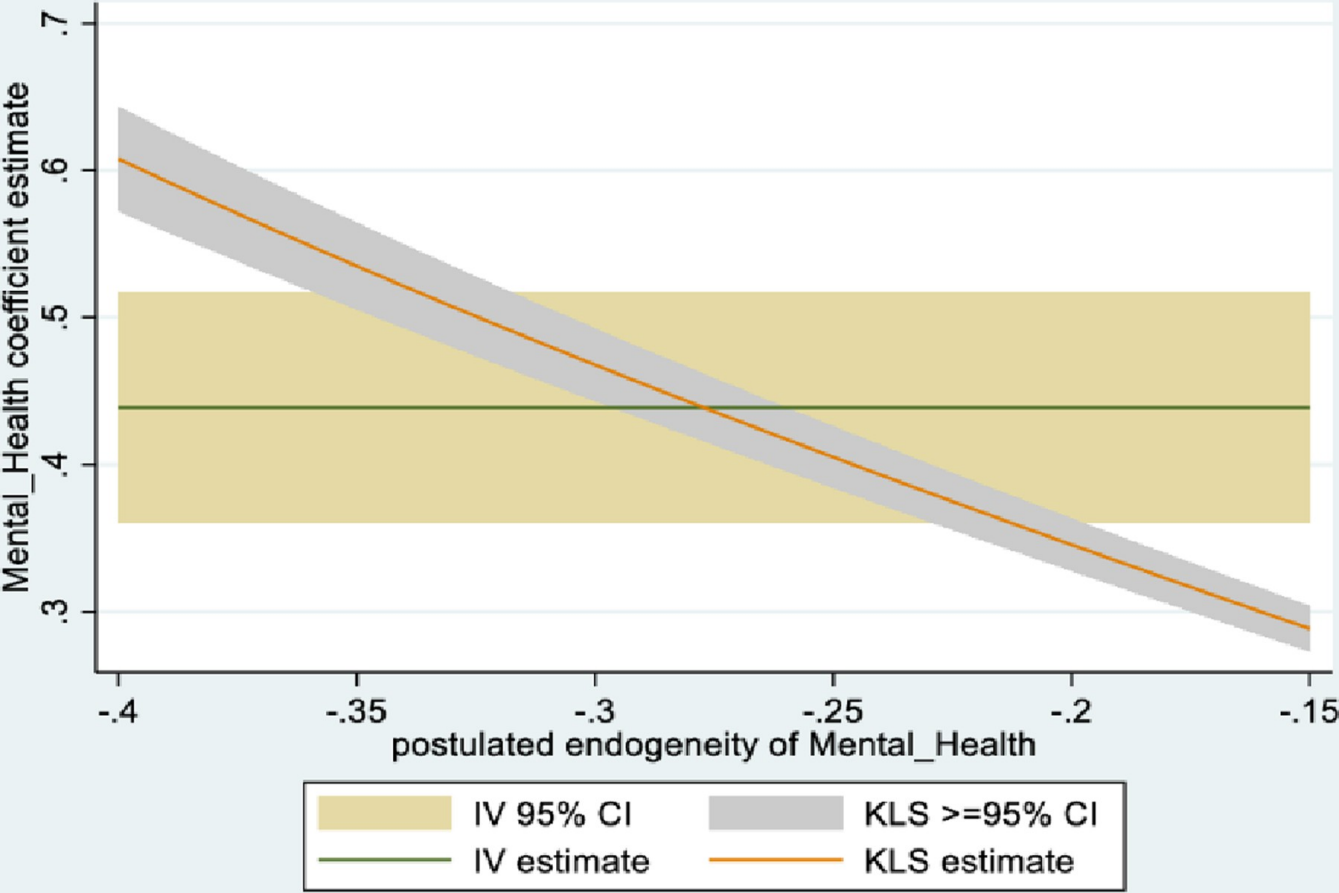
Model 2 KLS estimates: Females.

**Fig 8:**
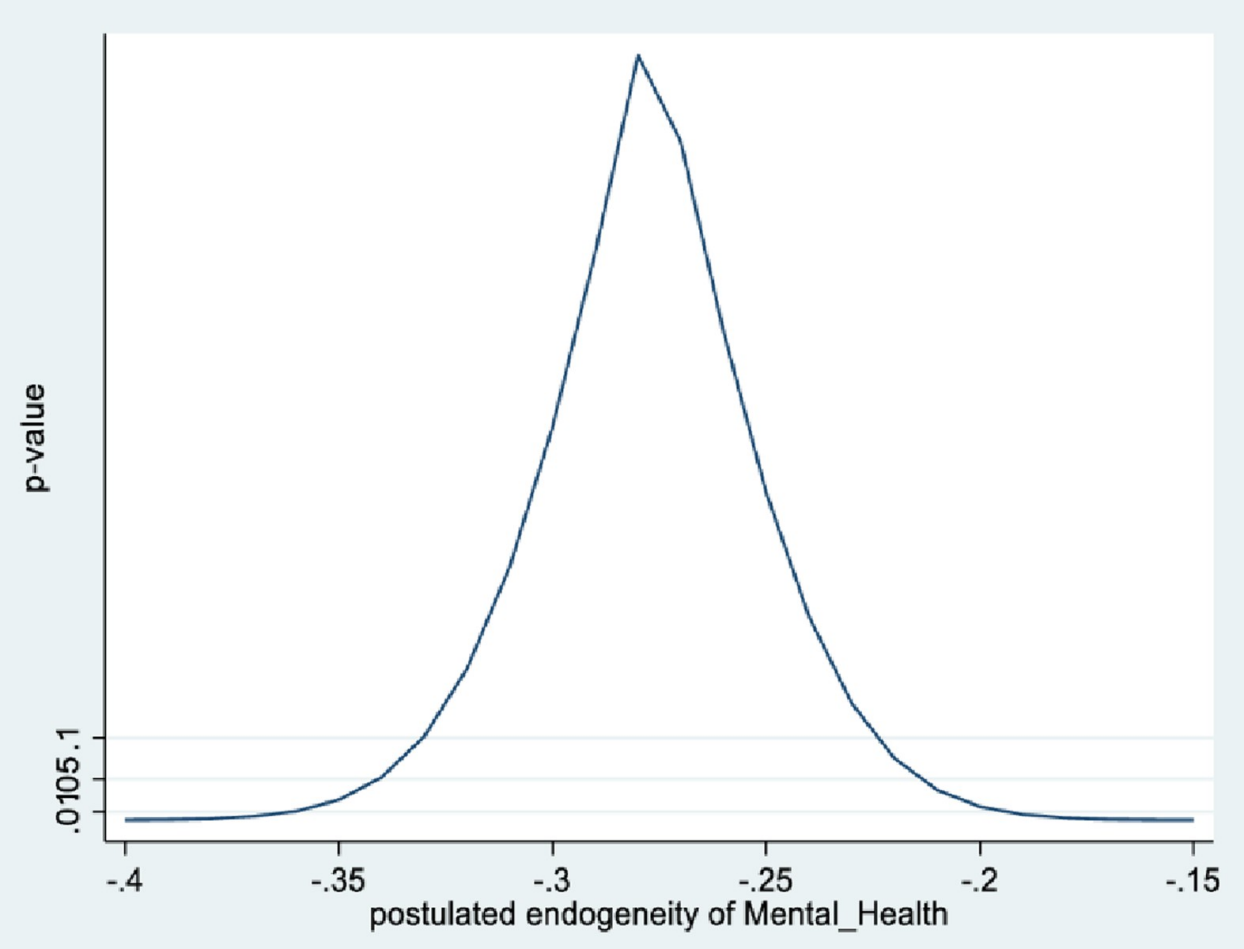
Model 2 exclusion restriction test: Females Endogeneity correlation point estimate: -0.32.

In the male sample, the joint null hypothesis that the instrument set is uncorrelated with the error term and that the excluded instruments are correctly excluded from the estimated equation is accepted at high p-values. Further evidence is given from KLS-based estimates and exclusion restrictions tests of each individual instrument (Figs 5–8). Using 95% confidence intervals of the degree of endogeneity compatible with the valid exclusion of the instrument, these intervals are [-0.43, -0.31] and [-0.53, -0.22] for the two instruments in the male regression. In the female regression, with one instrument, the 95% confidence interval for the degree of endogeneity compatible with the valid exclusion of the instrument is [-0.34, -0.21]. Finally, the first stage F-values of excluded instruments in the IV regressions (47 and 90 for males and females, respectively) indicate that the instrument set is not weak.

Overall, the IV and KLS estimates from the two models tell a consistent story. In the direction from social connectedness to mental health, evidence of endogeneity of social connectedness perceptions is weak, especially in the male sample; hence, after accounting for endogeneity biases the effect of social connectedness on mental health is modest and of similar magnitude for male and female participants. However, in the direction from mental health to social connectedness, accounting for biases associated with the endogeneity of mental health perceptions is important. Comparing effect sizes, the dominant effect is from mental health to social connectedness.

### 3.3 Other covariates

Looking at significant associations between covariates and the outcome in the two models, in Model 1 the larger associations are found between better overall health and better mental health. The estimated effect sizes are large, with more than one SD better mental health when the comparison is between assessing health as excellent vs. poor. On the other hand, in the opposite direction, better overall health is only weakly associated with social connectedness perceptions. Looking at other covariates, among student demographic and family characteristics, being of immigrant background is negatively associated with social connectedness (and more so for female participants). Reporting more interest in ICT is negatively associated with mental health perceptions; however, the opposite is the case for its association with social connectedness. Among psychological constructs, reporting a stronger fear of failure is negatively associated with mental health perceptions. On the other hand, reporting higher resilience associated with better social connectedness, but no association between resilience and mental health was found. Finally, among school-level variables, the association between male students attending a single-sex school and both mental health and social connectedness perceptions is negative, but this is not the case for females for either of the two outcomes.

### 3.4 Further analysis

#### 3.4.1 Assuming imperfect instruments

I derived IV bounds on estimates when assumptions underlying instrument validity do not hold precisely, i.e., relaxing the zero-covariance assumption between the instrument and the error term, using the Nevo and Rosen [38] approach. Given an endogenous covariate of interest, x, and an instrument, z, instead of assuming that ρ_zε_ = 0, it is assumed that the instrument has (weakly) the same direction of correlation with the omitted error term as the endogenous covariate (i.e., ρ_xε_ρ_zε ≥_ 0). If the instrument is less endogenous than the endogenous covariate of interest (i.e., ρ_xε_ ≥ ρ_zε_), then this amounts to essentially considering an “imperfect” instrumental variable.

Bounds from IV estimation assuming imperfect instruments (derived using Stata’s *imperfectiv* module by Clarke and Matta [40] are given in Tables 4 and 5. As expected, the uncertainty associated with the bounds is larger. In Model 1, the lower bound CI includes the OLS estimates. This is not surprising, given that the evidence of endogeneity of the social connectedness composite is weak. In Model 2, the lower bound CI excludes the OLS estimates for both male and female participants; however, the overall 95% CI of estimates are wider, i.e., [0.17, 0.82] for males (compared to [0.28, 0.88] in the main results), and [0.11, 0.62] for females (compared to [0.19, 0.66]), due to wider lower bounds. Overall, the implications of findings regarding the relative size of effect estimates between Model 1 and Model 2 strongly support that the dominant effect is in the direction from mental health to social connectedness.

**Table 4:** Nevo and Rosen (2008)’s Imperfect IV bounds–Model 1.

Outcome: Mental Health	Social Connectedness: Lower bound CI	Social Connectedness: Lower bound estimate	Social Connectedness: Upper bound estimate	Social Connectedness: Upper bound CI
Males	0.075	0.228	-	-
Females	0.047	0.308	-	-

Note: Given that the correlation between the endogenous variable and the imperfect instrument is positive, only one-sided intervals are reported.

**Table 5:** Nevo and Rosen (2008)’s Imperfect IV bounds–Model 2.

Outcome: Social Integration	Social Integration: Lower bound CI	Social Integration: Lower bound estimate	Social Integration: Upper bound estimate	Social Integration: Upper bound CI
Males	[0.170	(0.230	0.549)	0.819]
Females	[0.105	(0.148	0.418)	0.619]

#### 3.4.2 Sensitivity to instrument choice in Model 2

**Fig 9:**
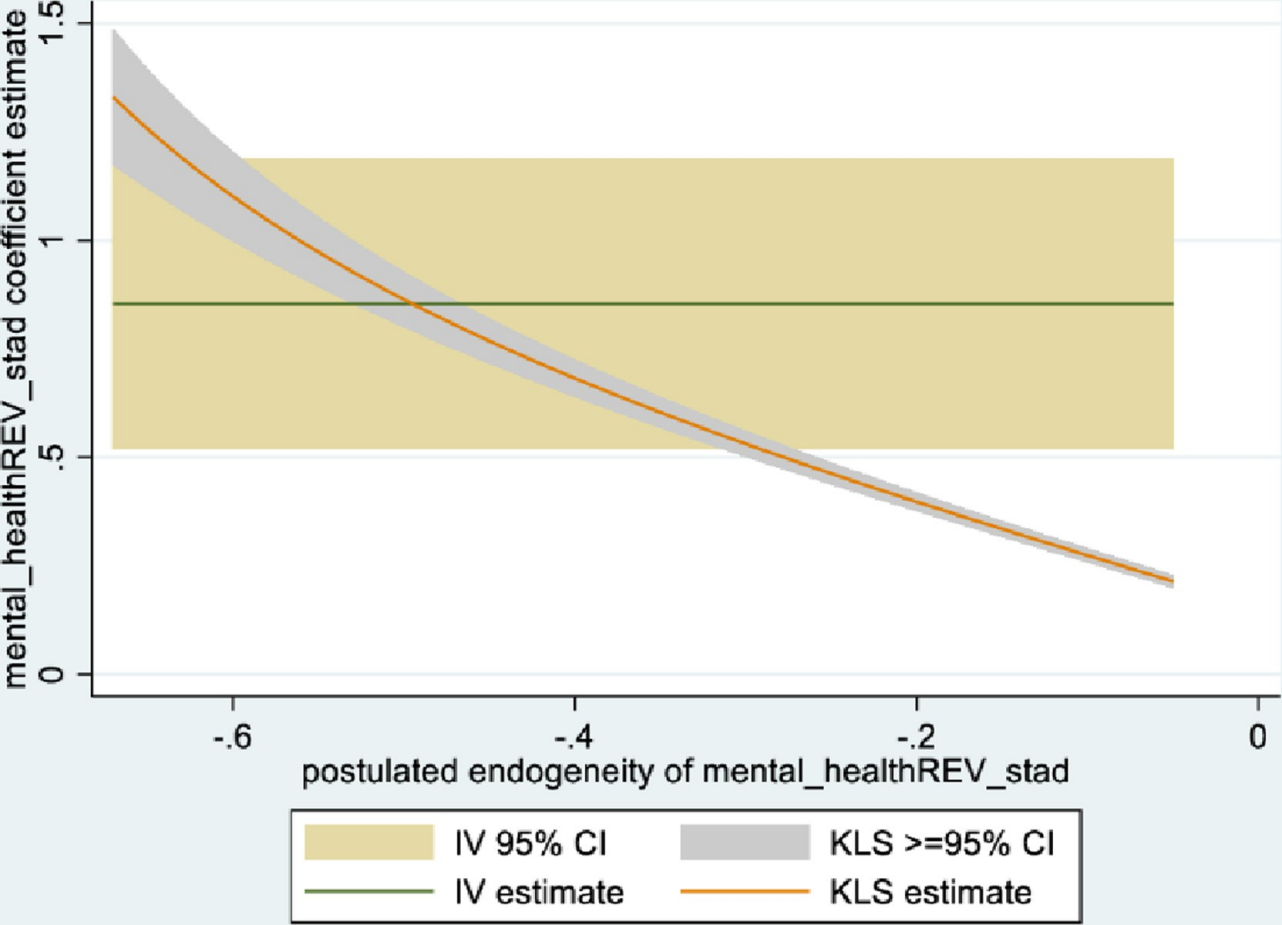
Model 2 KLS estimates with alternative instrument: Males.

**Fig 10:**
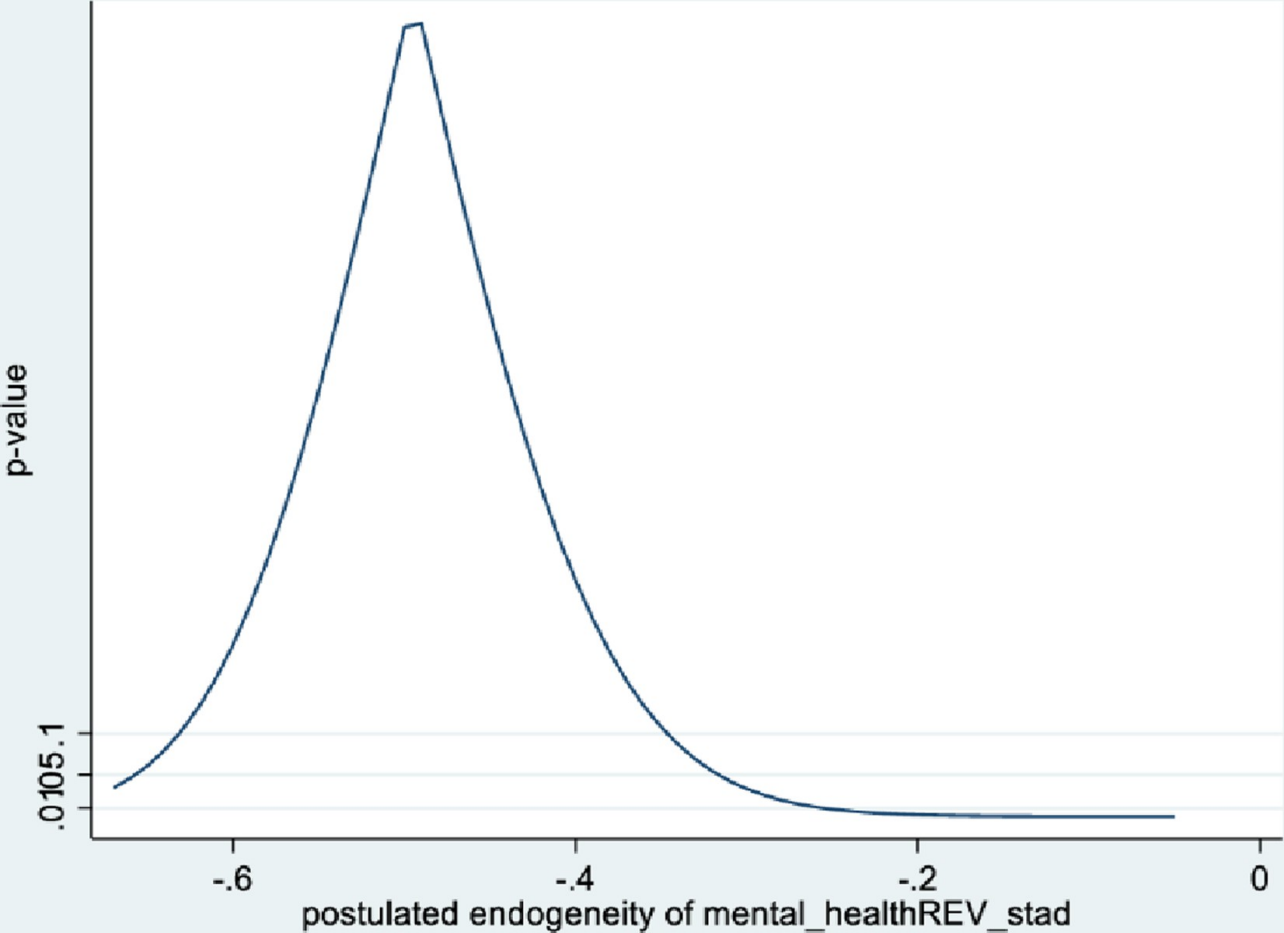
Model 2 exclusion restriction test with alternative instrument: Males. Endogeneity correlation point estimate: -0.36.

**Fig 11:**
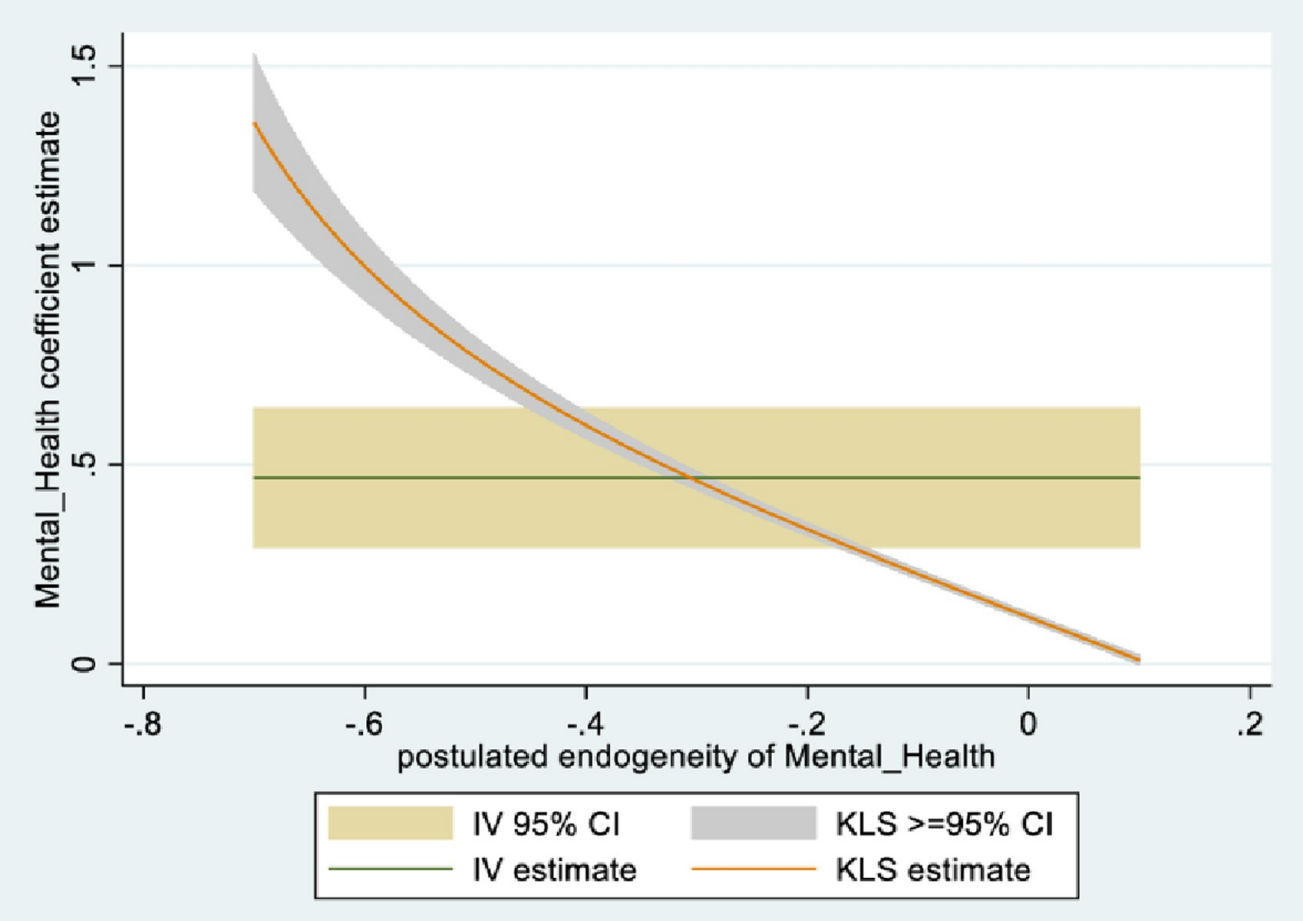
Model 2 KLS estimates with alternative instrument: Females.

**Fig 12:**
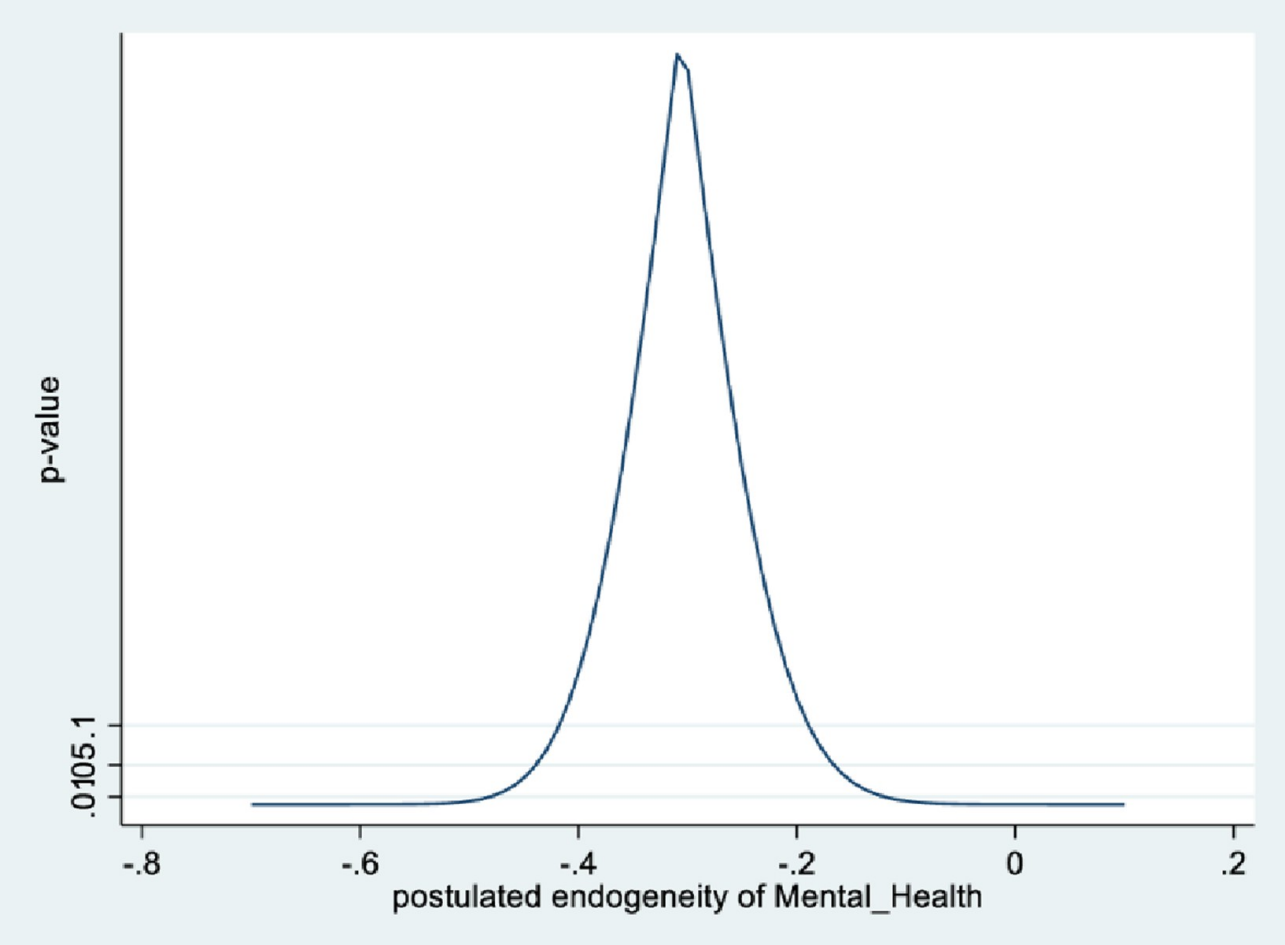
Model 2 exclusion restriction test with alternative instrument: Females. Endogeneity correlation point estimate: -0.39.

**Fig 13:**
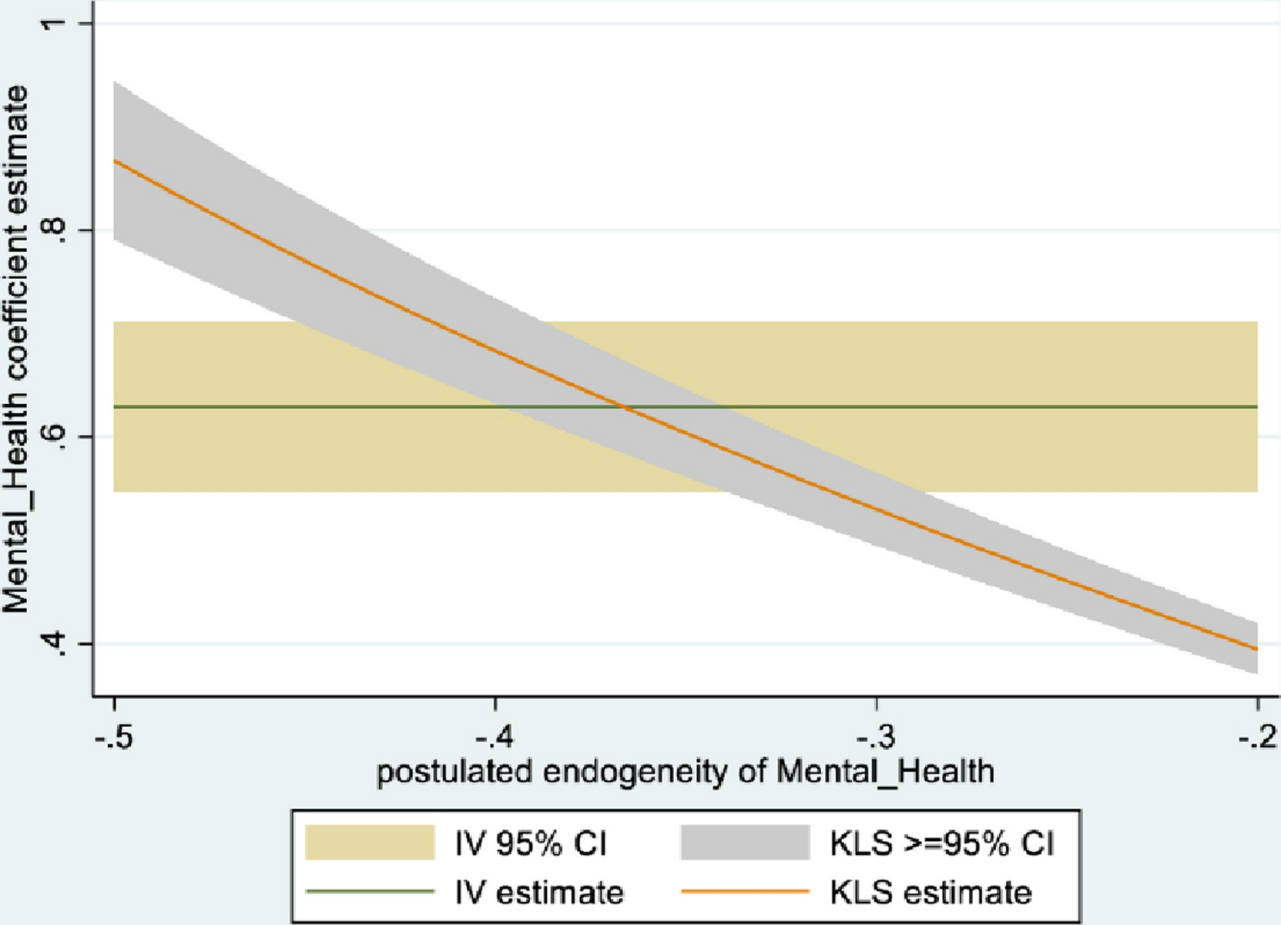
Model 2 KLS estimates with combination of instruments: Males.

**Fig 14:**
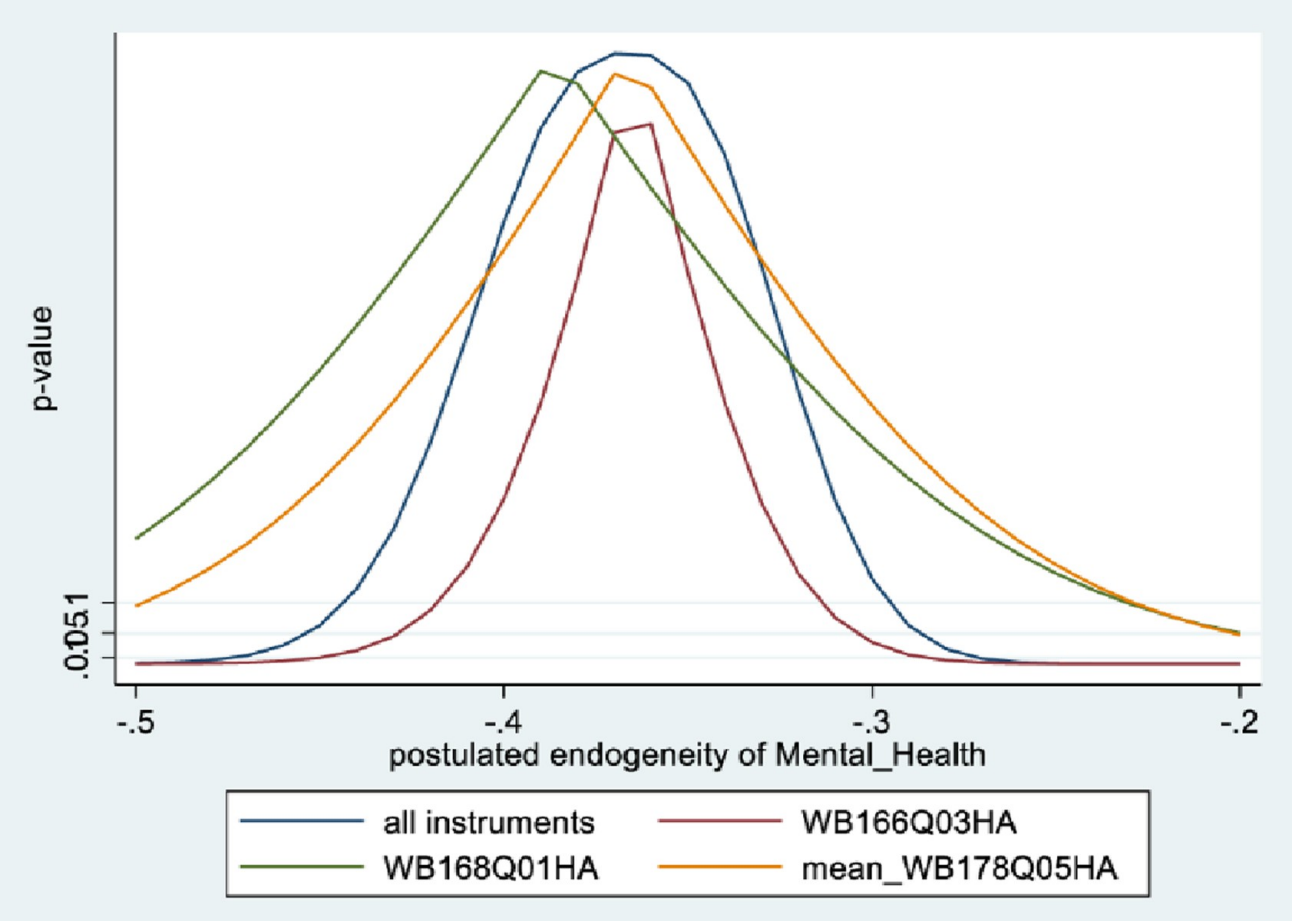
Model 2 exclusion restriction test with combination of instruments: Males. Endogeneity correlation point estimate: -0.36.

**Fig 15:**
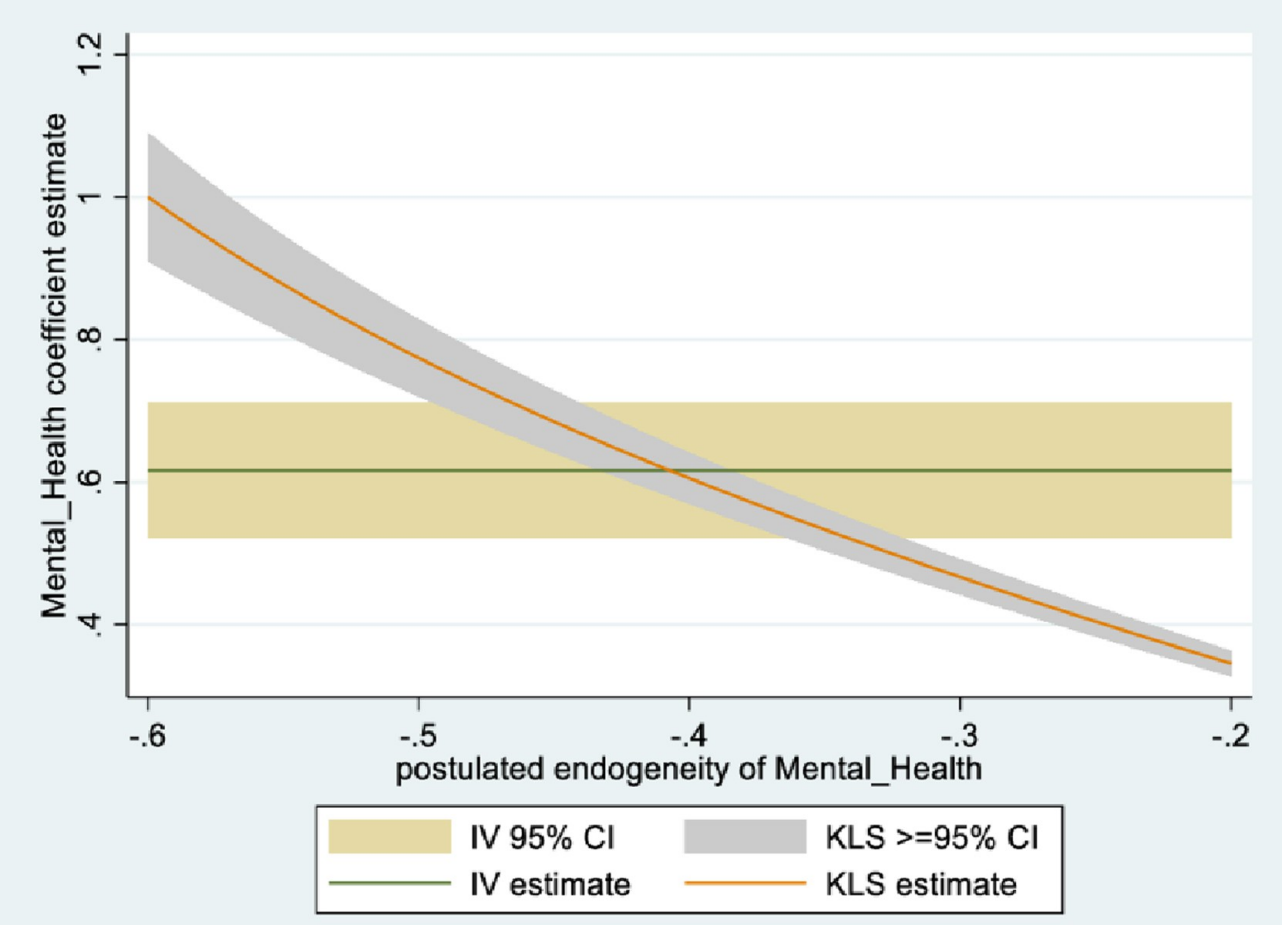
Model 2 KLS estimates with combination of instruments: Females.

**Fig 16:**
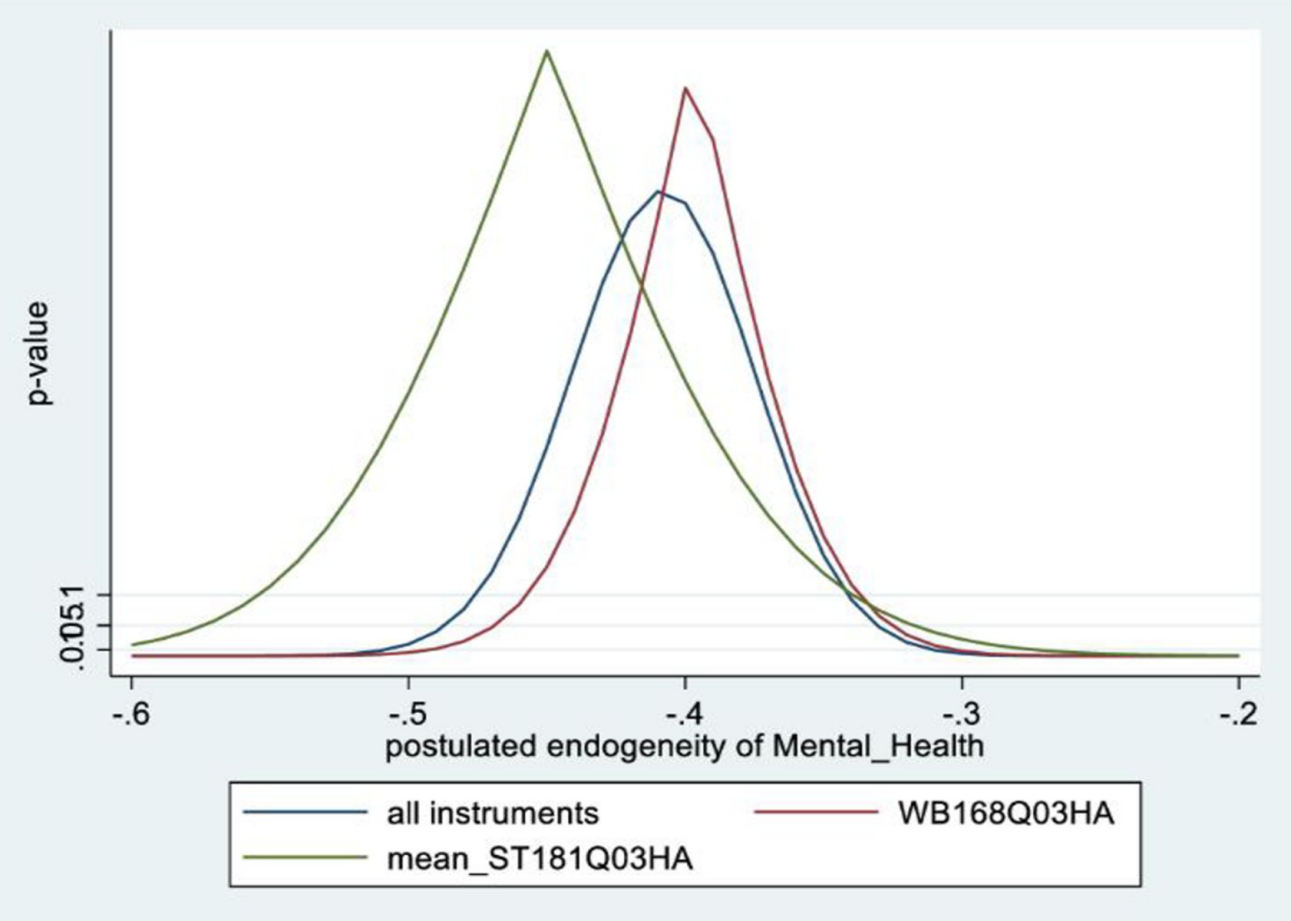
Model 2 exclusion restriction test with combination of instruments: Females. Endogeneity correlation point estimate: -0.44.

The alternative instruments used in IV estimation in Model 2 are school-level instruments. For males, it is the school mean for agreeing with the statement: ‘*Yesterday [a typical day] I had enough energy*’; for females, it is the school mean response (strongly disagree, to strongly agree) to the statement: ‘*it is important for me to perform better than other people*.’ As expected, the first is positively correlated with individual students’ mental health (i.e., less mental health-related complaints), while the second (i.e., more competitive school environment), is negatively correlated with individual students’ mental health. Figs 9–12 contain the information from testing the exclusion restrictions (95% CI of the degree of endogeneity of the mental health composite compatible with valid exclusion) for the alternative instrument. Figs 13–16 contain the corresponding information when the alternative instrument is combined with the main instrument/s (i.e., those behind the results in Table 3).

Table 6 contains the IV estimates using the alternative instrument (column 1) and the combination of the main and the alternative instrument (column 2). In column 1, the point estimates of the effect of perceived mental health on perceived social connectedness (β = 0.54 for males and 0.48 for females), are essentially identical to those in Table 3. However, they are substantially less precise (95% CI: [0.05, 1.03] for males and [-0.08, 1.04] for females). In column 2, the combination of three instruments for males and two instruments for females results in more precise point estimates, which nevertheless present the same picture (β = 57; 95% CI = [0.38, 0.76] for males β = 0.53; 95% CI = [0.30, 0.75] for females). Based on the overidentification tests of all instruments (J-test), the hypothesis that the set of additional instruments is exogenous is accepted at high p-values for both males and females.

**Table 6:** From mental health to social integration: Alternative instruments.

	(1)		(2)	
Outcome: Social Integration (stand. index)	MALES	FEMALES	MALES	FEMALES
	IV	IV	IV	IV
**Mental Health (stand. index)**	**0.544**	0.481	**0.568**	**0.527**
	(0.252)	(0.289)	(0.098)	(0.113)
Relevance of excluded instrument				
F-statistic for excluded instruments	18.0	17.1	40.8	41.6
Overidentification test of all instruments				
Hansen’s J statistic	-	-	0.389	0.032
[p-value]			[0.823]	[0.858]
Test of endogeneity of mental health				
Chi-sq. statistic	2.38	2.04	20.1	17.9
[p-value]	[0.123]	[0.153]	[0.000]	[0.000]
**N**	23,506	25,761	20,607	23,401

(1): Instrument for males: School mean of: *Did you have enough energy to get things done yesterday*? ‐ *Yes*

Instrument for females: School mean of: *It is important for me to perform better than other people ‐ Agree*

(2): Instrument set for males: Combination of three instruments (main + alternative instrument).

Instrument set for females: Combination of two instruments (main + alternative instrument).

#### 3.4.3 Sensitivity of results to model specification

Some of the covariates in the models other than the covariates of interest, are based on students’ perceptions about themselves, hence potentially endogenous. I re-estimated the models with other potentially endogenous covariates omitted, keeping only demographic, individual student, and school characteristics. From the estimation results (reported in Tables 7 and 8), the overall picture did not change (large effects from mental health to social connections, modest effect estimates from social connections to mental health). However, effect estimates are 15–20% higher than when controlling for the full set of observable covariates, given that the coefficient estimates of interest now partially reflect the effect of omitted characteristics on outcome. Furthermore, the validity of exclusion restrictions is accepted at lower p-values compared to the model with the full set of controls. These findings seem to support suggestions that the assumptions related to identification may be more plausible after conditioning on covariates and that instruments are sometimes valid only after conditioning for certain covariates [41, 42].

**Table 7:** From social integration to mental health: IV estimates omitting other potential endogenous regressors.

Outcome: Mental Health (stand. index)	MALES	FEMALES
Social Connectedness (stand. index)	**0.268** (0.061)	**0.321** (0.094)

**Table 8:** From mental health to social integration: IV estimates omitting other potential endogenous regressors.

Outcome: Social Integration (stand. index)	MALES	FEMALES
Mental Health (stand. index)ealth	**0.695** (0.088)	**0.491** (0.082)

## 4. Discussion and implications

Research focusing on the bidirectional relationship between social connectedness and health, including mental health, of adolescents is scarce, as most studies have been on adults and few studies considered a bidirectional relationship. Some of the existing studies exploited the availability of longitudinal data to provide evidence of the *existence* of a causal relationship, either from social connectedness to health or establish a bidirectional relationship. However, while acknowledging potential biases in estimated effects as a limitation, these studies did not account for such biases in their methodological approach. Furthermore, these studies account for only a relatively small set of other covariates. Finally, the use of large, multi-country samples is virtually absent. A frequent finding in the related empirical literature either on adults or adolescents is that the strongest path is from connectedness to health/mental health. Studies generally report that standardized effect sizes are modest.

The quasi-experimental approach implemented in this study intended to assess the importance of endogeneity and associated biases in each direction of the relationship, assess the size of the bias-corrected effects in each direction, and compare the findings to those prevalent in the literature. Addressing RQ1, the findings indicate that accounting for endogeneity biases is important. Such biases are large in the direction from mental health to social connectedness for both males and females. In our case, the strongly negative endogeneity correlations are associated with a downward bias. Addressing RQ2, the findings point to large effect estimates from mental health to social connectedness for both male and female participants. For a student reporting social connectedness at around the middle of the percentile distribution of the social connectedness composite, the standardized effect size estimated (at about 0.4–0.6 SD increase in social connectedness) corresponds to about 20 points higher in the distribution of the social connectedness composite. The main finding, therefore, is that the dominant effect is from mental health to social connectedness and effect sizes are large and of similar magnitude by gender. These findings are very different than those reported in the literature, which either assumes only one direction in the relationship (while acknowledging the possibility of presence of effects in the opposite direction) or report a dominant effect from social connectedness to mental health. Finally, addressing RQ3, modest gender differences in effect size were uncovered in Model 1; otherwise, the implications of the findings apply to both male and female participants.

### 4.1 Implications and policy relevance

The clear implication of the main finding is that the predominance of findings supporting the dominance of the effect in the direction from social connectedness to mental health may be due to failure to account for the influence of unobservables. The intuition for the importance of adolescent mental health in maintaining social relationships is that adolescent students with mental health symptoms can progressively become socially isolated because of negative experiences when interacting with others, as well as negative reactions of others. Mental health can also affect how social situations are perceived which can, in turn, affect one’s behaviour. Given the predominance of earlier findings of a dominant effect in the direction from social connectedness to mental health, policies have been increasingly emphasizing the importance of social relationships for mental health, including adolescent mental health. However, in medical and psychiatric settings, patients’ social functioning is rarely the point of intervention; instead, it is often considered an indicator of impaired mental health [43, 18].

The policy relevance of the findings in this study is that adolescent mental health should be the primary focus of interventions, i.e., identifying and treating mental health symptoms as a primary intervention and as a precursor to improving the social connectedness of adolescents. Schwartz and Litwin [44] also reported dominance of the direction from mental health to social connections, but in older adults, refer to the possibility of a “vicious cycle” in which those in worse mental state may end up with a less supportive social environment, which in turn may result in a deterioration of mental health. This is also relevant to adolescents, given that adolescence and the onset of puberty is a critical period for neurological, social, and cognitive development, potentially associated with symptoms of psychological nature. Onset of mental health symptoms may result in social withdrawal and further mental health impairment.

### 4.2 Strengths and limitations

I outlined and implemented a quasi-experimental approach to derive effect estimates when the covariate of interest is potentially endogenous. This is often the case with covariates derived from survey participants’ perceptions or when using psychological constructs. As was the case of this study, biases associated with the endogeneity of covariates of interest can be important when looking for answers to research questions.

This study has limitations. The data sample used for analysis comprises nine countries, i.e., the countries that participated in the wellbeing module of PISA 2018. Hence, the findings may not be generalizable; had the estimation sample been larger, findings may have been somewhat different. Finally, it would be preferable to implement the quasi-experimental approach used in this study with longitudinal data.
